# Selecting additional tag SNPs for tolerating missing data in genotyping

**DOI:** 10.1186/1471-2105-6-263

**Published:** 2005-11-01

**Authors:** Yao-Ting Huang, Kui Zhang, Ting Chen, Kun-Mao Chao

**Affiliations:** 1 Department of Computer Science and Information Engineering, National Taiwan University, Taipei, Taiwan; 2 Graduate Institute of Networking and Multimedia, National Taiwan University, Taipei, Taiwan; 3 Section on Statistical Genetics, Department of Biostatistics, University of Alabama at Birmingham, USA; 4 Department of Biological Sciences, University of Southern California, Los Angeles, CA 90089, USA

## Abstract

**Background:**

Recent studies have shown that the patterns of linkage disequilibrium observed in human populations have a block-like structure, and a small subset of SNPs (called tag SNPs) is sufficient to distinguish each pair of haplotype patterns in the block. In reality, some tag SNPs may be missing, and we may fail to distinguish two distinct haplotypes due to the ambiguity caused by missing data.

**Results:**

We show there exists a subset of SNPs (referred to as robust tag SNPs) which can still distinguish all distinct haplotypes even when some SNPs are missing. The problem of finding minimum robust tag SNPs is shown to be NP-hard. To find robust tag SNPs efficiently, we propose two greedy algorithms and one linear programming relaxation algorithm. The experimental results indicate that (1) the solutions found by these algorithms are quite close to the optimal solution; (2) the genotyping cost saved by using tag SNPs can be as high as 80%; and (3) genotyping additional tag SNPs for tolerating missing data is still cost-effective.

**Conclusion:**

Genotyping robust tag SNPs is more practical than just genotyping the minimum tag SNPs if we can not avoid the occurrence of missing data. Our theoretical analysis and experimental results show that the performance of our algorithms is not only efficient but the solution found is also close to the optimal solution.

## Background

In recent years, *Single Nucleotide Polymorphisms *(SNPs) have become the preferred marker for association studies of genetic diseases or traits. A set of linked SNPs on one chromosome is called a *haplotype*. Recent studies have shown that the patterns of *Linkage Disequilibrium *(LD) observed in human populations have a block-like structure [4,13]. The chromosome recombination only takes place at some low LD regions called recombination hotspots. The high LD region between these hotspots is often referred to as a "haplotype block." Within a haplotype block, there is little or even no recombination occurred, and the SNPs in the block tend to be inherited together. Due to the low haplotype diversity within a block, the information carried by these SNPs is highly redundant. Thus, a small subset of SNPs (called "tag SNPs") is sufficient to distinguish each pair of patterns in the block [7,13,17-19]. Haplotype blocks with corresponding tag SNPs are quite useful and cost-effective for association studies as it does not require genotyping all SNPs. Many studies have tried to find the minimum set of tag SNPs in a haplotype block. In a large-scale study of human Chromosome 21, Patil *et al*. [13] developed a greedy algorithm to partition the haplotypes into 4,135 blocks with 4,563 tag SNPs. Zhang *et al*. [17-19] used a dynamic programming approach to reduce the numbers of blocks and tag SNPs to 2,575 and 3,562, respectively. Bafna *et al*. [1] showed that the problem of minimizing tag SNPs is NP-hard and gave efficient algorithms for special cases of this problem. 

**Figure 1:**
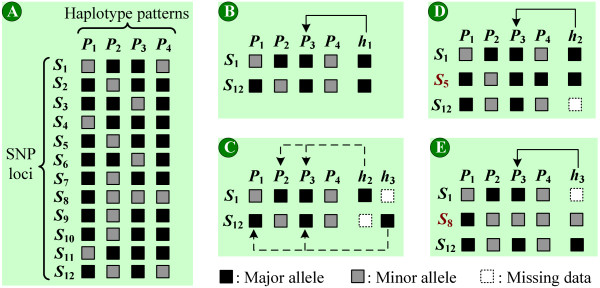
**The influence of missing data and auxiliary tag SNPs**. (A) A haplotype block defined by 12 SNPs and 4 haplotype patterns. Each column represents a haplotype pattern and each row represents a SNP locus. The black and grey boxes stand for the major and minor alleles at each SNP locus, respectively. (B) Tag SNPs genotyped without missing data. (C) Tag SNPs genotyped with missing data. (D) The auxiliary tag SNP *S*_5 _for *h*_2_. (E) The auxiliary tag SNP *S*_8 _for *h*_3_.

In reality, a SNP may not be genotyped and considered to be missing data (i.e., we fail to obtain the allele configuration of the SNP) if it does not pass the threshold of data quality [13,16,19,20]. These missing data may cause ambiguity when using the minimum set of tag SNPs to distinguish an unknown haplotype sample. Figure 1 illustrates the influence of missing data when identifying haplotype samples. In this figure, a haplotype block (see Figure 1 (A)) defined by 12 SNPs and 4 haplotype patterns is presented (from the public haplotype data of human Chromosome 21 [13]). We follow the same assumption as previous studies that all SNPs are diallelic (i.e., taking on only two values) [1,13]. Suppose we select SNPs *S*_1 _and *S*_12 _as tag SNPs. The haplotype sample *h*_1 _is identified as haplotype pattern *P*_3 _unambiguously (see Figure 1 (B)). Consider haplotype samples *h*_2 _and *h*_3 _with one missing tag SNP (see Figure 1 (C)). *h*_2 _can be identified as haplotype patterns *P*_2 _or *P*_3_, and *h*_3 _can be identified as *P*_1 _or *P*_3_. As a result, these missing tag SNPs result in ambiguity when distinguishing unknown haplotype samples.

Although we can not avoid the occurrence of missing data, the remaining SNPs within the haplotype block may provide abundant information to resolve the ambiguity. For example, if we re-genotype an additional SNP *S*_5 _for *h*_2 _(see Figure 1 (D)), *h*_2 _is identified as haplotype pattern *P*_3 _unambiguously. On the other hand, if SNP *S*_8 _is re-genotyped (see Figure 1 (E)), *h*_3 _is also identified unambiguously. These additional SNPs are referred to as "auxiliary tag SNPs," which can be found from the remaining SNPs in the block and are able to resolve the ambiguity caused by missing data.

**Figure 2:**
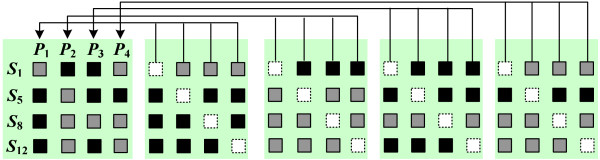
**The robust tag SNPs**. A set of robust tag SNPs for tolerating one missing tag SNP.

Alternatively, instead of re-genotyping auxiliary tag SNPs whenever encountering missing data, we work on a set of SNPs which is not affected by the occurrence of missing data. Figure 2 illustrates a set of SNPs which can tolerate one missing SNP. Suppose we select SNPs *S*_1_, *S*_5_, *S*_8_, and *S*_12 _to be genotyped. Note that no matter which SNP is missing, each of the 16 missing patterns can be distinguished by the remaining three SNPs. Therefore, all haplotype samples with one missing SNP can still be identified unambiguously. We refer to these SNPs as "robust tag SNPs," which are able to tolerate a number of missing data. The important feature of robust tag SNPs is that although they consume more SNPs than the "tag SNPs" defined in previous studies, they guarantee that all haplotype samples with a number of missing data can be distinguished unambiguously. When the occurrence of missing data is frequent, the cost of re-genotyping processes can be reduced by robust tag SNPs.

This paper focuses on the problem of finding robust tag SNPs to tolerate a number of missing data. Throughout this paper, we denote *m *as the maximum number of missing SNPs to be tolerated, which corresponds to different missing rates in different genotyping experiments. And we wish to find a minimum set of robust tag SNPs which can distinguish each pair of haplotypes even when up to *m *SNPs are missing. We assume that the haplotype phases and block partition are available as the input. Numerous methods have been developed to infer haplotypes from genotype data [12,14,15]. Several algorithms have also been proposed to find the block partition [4,13,17]. The problem of finding minimum robust tag SNPs is shown to be NP-hard (See Theorem 1). To find robust tag SNPs efficiently, we propose two greedy algorithms and one linear programming (LP) relaxation algorithm. The proposed algorithms have been implemented and tested on a variety of simulated and empirical data. We also analyze the efficiency and solutions of these algorithms. An algorithm for finding auxiliary tag SNPs is described assuming robust tag SNPs have been computed in advance.

## Results

We propose two greedy algorithms which select the robust tag SNPs one by one in different greedy manners. In addition, we reformulate this problem as an integer programming problem and design an LP-relaxation algorithm to solve this problem. The greedy and LP-relaxation algorithms are able to find solutions within factors of (*m *+ 1) lnK(K−1)2
 MathType@MTEF@5@5@+=feaafiart1ev1aaatCvAUfKttLearuWrP9MDH5MBPbIqV92AaeXatLxBI9gBaebbnrfifHhDYfgasaacH8akY=wiFfYdH8Gipec8Eeeu0xXdbba9frFj0=OqFfea0dXdd9vqai=hGuQ8kuc9pgc9s8qqaq=dirpe0xb9q8qiLsFr0=vr0=vr0dc8meaabaqaciGacaGaaeqabaqabeGadaaakeaacqqGSbaBcqqGUbGBdaWcaaqaaiabdUealjabcIcaOiabdUealjabgkHiTiabigdaXiabcMcaPaqaaiabikdaYaaaaaa@363F@, ln((m+1)K(K−1)2)
 MathType@MTEF@5@5@+=feaafiart1ev1aaatCvAUfKttLearuWrP9MDH5MBPbIqV92AaeXatLxBI9gBaebbnrfifHhDYfgasaacH8akY=wiFfYdH8Gipec8Eeeu0xXdbba9frFj0=OqFfea0dXdd9vqai=hGuQ8kuc9pgc9s8qqaq=dirpe0xb9q8qiLsFr0=vr0=vr0dc8meaabaqaciGacaGaaeqabaqabeGadaaakeaacqqGSbaBcqqGUbGBcqqGOaakcqqGOaakcqWGTbqBcqGHRaWkcqaIXaqmcqGGPaqkdaWcaaqaaiabdUealjabcIcaOiabdUealjabgkHiTiabigdaXiabcMcaPaqaaiabikdaYaaacqGGPaqkaaa@3CD6@, and *O*(*m *ln *K*) of the optimal solution respectively, where *m *is the maximum number of missing SNPs allowed and *K *is the number of haplotype patterns in the block.

We have implemented the first and second greedy algorithms in JAVA [see Additional files 1 and 2]. The LP-relaxation algorithm has been implemented in Perl [see Additional file 3], where the LP problem is solved via a program called "lp_solve" [11]. The LP-relaxation algorithm is a randomized method. Thus, this program is repeated for 10 times to explore different solutions and the best solution among them is chosen as the output.

In order to evaluate the solutions and efficiency of our algorithms, we also implement a program in JAVA (referred to as "OPT") which uses a brute force method to find the optimal solution. For a given data set of *N *SNPs, the OPT program examines all possible solutions (i.e., all subsets of (N1)
 MathType@MTEF@5@5@+=feaafiart1ev1aaatCvAUfKttLearuWrP9MDH5MBPbIqV92AaeXatLxBI9gBaebbnrfifHhDYfgasaacH8akY=wiFfYdH8Gipec8Eeeu0xXdbba9frFj0=OqFfea0dXdd9vqai=hGuQ8kuc9pgc9s8qqaq=dirpe0xb9q8qiLsFr0=vr0=vr0dc8meaabaqaciGacaGaaeqabaqabeGadaaakeaadaqadaqaauaabeqaceaaaeaacqWGobGtaeaacqaIXaqmaaaacaGLOaGaayzkaaaaaa@3059@, (N2)
 MathType@MTEF@5@5@+=feaafiart1ev1aaatCvAUfKttLearuWrP9MDH5MBPbIqV92AaeXatLxBI9gBaebbnrfifHhDYfgasaacH8akY=wiFfYdH8Gipec8Eeeu0xXdbba9frFj0=OqFfea0dXdd9vqai=hGuQ8kuc9pgc9s8qqaq=dirpe0xb9q8qiLsFr0=vr0=vr0dc8meaabaqaciGacaGaaeqabaqabeGadaaakeaadaqadaqaauaabeqaceaaaeaacqWGobGtaeaacqaIYaGmaaaacaGLOaGaayzkaaaaaa@305B@, ..., and (NN)
 MathType@MTEF@5@5@+=feaafiart1ev1aaatCvAUfKttLearuWrP9MDH5MBPbIqV92AaeXatLxBI9gBaebbnrfifHhDYfgasaacH8akY=wiFfYdH8Gipec8Eeeu0xXdbba9frFj0=OqFfea0dXdd9vqai=hGuQ8kuc9pgc9s8qqaq=dirpe0xb9q8qiLsFr0=vr0=vr0dc8meaabaqaciGacaGaaeqabaqabeGadaaakeaadaqadaqaauaabeqaceaaaeaacqWGobGtaeaacqWGobGtaaaacaGLOaGaayzkaaaaaa@308E@). The minimum subset of SNPs that can tolerate *m *missing SNPs is chosen as the output. Due to the NP-hardness of this problem, the OPT program fails to output the optimal solution within a reasonable period of time in many data sets. As a consequence, we skip some impossible solution space to speed up this program by the following two observations: (1) the solutions with less than or equal to *m *SNPs are the impossible ones since *m *SNPs might be missing; and (2) for a data set containing *K *haplotype patterns, the minimum number of SNPs required to distinguish each of them is at least log *K *(see Lemma 2). As a result, we can examine the possible solutions only for subsets of (Nm+log⁡  K)
 MathType@MTEF@5@5@+=feaafiart1ev1aaatCvAUfKttLearuWrP9MDH5MBPbIqV92AaeXatLxBI9gBaebbnrfifHhDYfgasaacH8akY=wiFfYdH8Gipec8Eeeu0xXdbba9frFj0=OqFfea0dXdd9vqai=hGuQ8kuc9pgc9s8qqaq=dirpe0xb9q8qiLsFr0=vr0=vr0dc8meaabaqaciGacaGaaeqabaqabeGadaaakeaadaqadaqaauaabeqaceaaaeaacqWGobGtaeaacqWGTbqBcqGHRaWkcyGGSbaBcqGGVbWBcqGGNbWzcaaMc8UaaGPaVlabdUealbaaaiaawIcacaGLPaaaaaa@3A01@, (Nm+log⁡  K+1)
 MathType@MTEF@5@5@+=feaafiart1ev1aaatCvAUfKttLearuWrP9MDH5MBPbIqV92AaeXatLxBI9gBaebbnrfifHhDYfgasaacH8akY=wiFfYdH8Gipec8Eeeu0xXdbba9frFj0=OqFfea0dXdd9vqai=hGuQ8kuc9pgc9s8qqaq=dirpe0xb9q8qiLsFr0=vr0=vr0dc8meaabaqaciGacaGaaeqabaqabeGadaaakeaadaqadaqaauaabeqaceaaaeaacqWGobGtaeaacqWGTbqBcqGHRaWkcyGGSbaBcqGGVbWBcqGGNbWzcaaMc8UaaGPaVlabdUealjabgUcaRiabigdaXaaaaiaawIcacaGLPaaaaaa@3BD3@ ..., and (NN)
 MathType@MTEF@5@5@+=feaafiart1ev1aaatCvAUfKttLearuWrP9MDH5MBPbIqV92AaeXatLxBI9gBaebbnrfifHhDYfgasaacH8akY=wiFfYdH8Gipec8Eeeu0xXdbba9frFj0=OqFfea0dXdd9vqai=hGuQ8kuc9pgc9s8qqaq=dirpe0xb9q8qiLsFr0=vr0=vr0dc8meaabaqaciGacaGaaeqabaqabeGadaaakeaadaqadaqaauaabeqaceaaaeaacqWGobGtaeaacqWGobGtaaaacaGLOaGaayzkaaaaaa@308E@. By searching possible solutions from small subsets to large ones, the OPT program can stop and output the optimal solution immediately when a subset that can tolerate *m *missing SNPs is found.

### Results on simulated data

**Figure 3:**
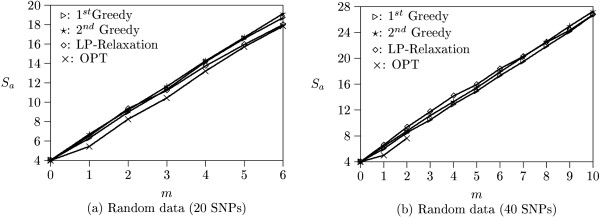
**Experimental results on random data**. (a) Results from data sets containing 10 haplotypes and 20 SNPs. (b) Results from data sets containing 10 haplotypes and 40 SNPs.

Theoretically, all SNPs will reach complete linkage equilibrium after sufficient chromosome recombination takes place. We first generate 100 data sets containing short haplotypes which simulate this bottleneck model [12,14,15]. Each data set consists of 10 haplotypes with 20 SNPs. These haplotypes are created by randomly assigning the major or minor alleles at each SNP locus. Let *m *be the number of missing SNPs allowed and *S*_*a *_be the average number of robust tag SNPs over 100 data sets. Figure 3 (a) plots *S*_*a *_with respect to *m *(roughly corresponding to SNP missing rates from 0% to 33%). When *m *= 0, all programs find the same number of SNPs as the optimal solution. The iterative LP-relaxation algorithm slightly outperforms the others as *m *increases. When *m *> 6, more than 20 SNPs are required to tolerate missing data. Thus, no data sets contain enough SNPs for solutions.

We then generate 100 data sets containing long haplotypes. Each data set is composed of 10 haplotypes with 40 SNPs. Figure 3 (b) illustrates the experimental results on these long data sets (corresponding to SNP missing rates from 0% to 37%). The optimal solutions for *m *> 2 can not be found by the OPT program within a reasonable period of time (after one week computation) and are not shown in this figure. It is because the possible solutions in long data sets are too large to enumerate. On the other hand, both greedy and iterative LP-relaxation algorithms run in polynomial time and always output a solution efficiently. In this experiment, both greedy algorithms slightly outperforms the iterative LP-relaxation algorithm. In addition, the number of missing SNPs allowed is larger than those in short data sets. For example, to tolerate 10 missing SNPs (i.e., *m *= 10), all programs output less than 28 SNPs. The remaining SNPs in each data set are still sufficient to tolerate more missing SNPs.

**Figure 4:**
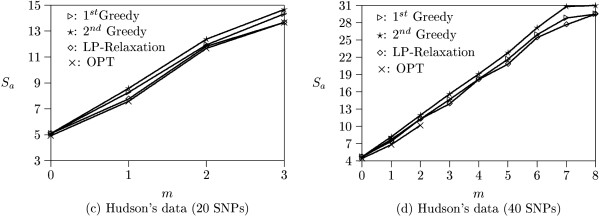
**Experimental results on Hudson's data**. (a) Results from data sets containing 10 haplotypes and 20 SNPs. (b) Results from data sets containing 10 haplotypes and 40 SNPs.

Hudson (2002) [10] provides a program which can simulate a set of haplotypes under the assumption of neutral evolution and uniformly distributed recombination rate using the coalescent model. We use Hudson's program to generate 100 short data sets with 10 haplotypes and 20 SNPs and 100 long data sets with 10 haplotypes and 40 SNPs. Figure 4 (a) shows the experimental results on Hudson's short data sets (corresponding to SNP missing rates from 0% to 23%). The number of missing SNPs allowed are less than that of random data. It is because Hudson's program generates coalescent haplotypes which are similar to each other. As a result, many SNPs can not be used to distinguish haplotypes and the amount of tag SNPs is inadequate to tolerate larger missing SNPs. In this experiment, we observe that the iterative LP-relaxation algorithm finds solutions quite close to the optimal solutions and slightly outperforms the other two algorithms.

Figure 4 (b) illustrates the experimental results on long data sets generated by Hudson's program (corresponding to SNP missing rates from 0% to 29%). The optimal solutions for *m *> 2 again can not be found by the OPT program within a reasonable period of time. In this experiment, the performance of the first greedy and iterative LP-relaxation algorithms are similar, and they slightly outperform the second greedy algorithm as *m *becomes large.

### Results on real data

We also test these programs on two real data sets: (1) public haplotype data of human Chromosome 21 released by Patil *et al*. [13]; and (2) a 500 KB region on human Chromosome 5q31 which may contain a genetic variant related to the Crohn disease by Daly *et al*. [4]. Patil's data include 20 haplotypes of 24,047 SNPs spanning over about 32.4 MB, which are partitioned into 4,135 haplotype blocks. By genotyping 103 SNPs with minor allele frequency at least 5%, Daly *et al*. partition the 500 KB region into 11 haplotype blocks. Each haplotype block in these real data sets contains different numbers of SNPs and haplotypes (e.g., from several SNPs to hundreds of SNPs). When *m *increases, some short blocks may not contain enough SNPs for tolerating missing data (e.g., *m *> the number of SNPs in a block). As a consequence, *S*_*a *_here stands for the average number of robust tag SNPs over those blocks still containing solutions.

**Figure 5:**
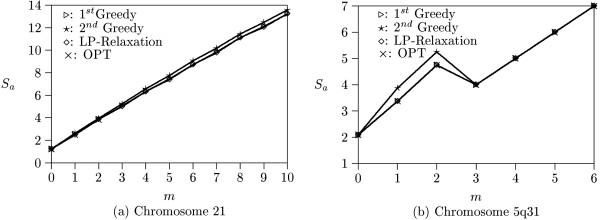
**Experimental results on real data**. (a) Results from Patil's Chromosome 21 data, (b) Results from Daly's Chromosome 5q31 data.

Figure 5 (a) shows the experimental results on Patil's 4,135 blocks. Because there are many long blocks in Patil's data (e.g., more than one hundred SNPs), the optimal solution for *m *> 2 can not be found within a reasonable period of time. The experimental result indicates that all algorithms find similar number of robust tag SNPs when *m *is small. The LP-relaxation algorithm slightly outperforms the others as *m *increases.

Figure 5 (b) illustrates the experimental results on Daly's 11 blocks. Because the haplotype blocks partitioned by Daly *et al*. are very short (e.g., most blocks contain less than 12 SNPs), all optimal solutions still can be found. The solutions found by each algorithm is almost the same as optimal solutions. Theoretically, *S*_*a *_should grow monotonically as *m *increases. But due to the small number of blocks in Daly's data set, *S*_*a *_does not grow smoothly when *m *increases from 2 to 3. To explain this phenomenon, we report the detailed result of the first greedy algorithm in Table 1. For each of the 11 blocks, the number of robust tag SNPs found with respect to different values of *m *is listed in the table. Note that as mentioned before, some blocks may not contain enough SNPs for tolerating large missing data as *m *increases. When *m *increases from 2 to 3, Blocks 4 and 10 (which consumes 8 and 5 SNPs) do not contain enough SNPs for a solution and are discarded. As a result, *S*_*a *_(for *m *= 3) is computed only using Blocks 1 and 2 and the value is lower than the previous one (i.e., from 4.75 to 4). This phenomenon is not shown in Figure 5 (a) because it is amortized by thousands of blocks in Patil's data set.

**Table 1:** The detailed result of first greedy algorithm on Daly's 11 blocks.

Block ID	1	2	3	4	5	6	7	8	9	10	11	*S*_*a*_
*m *= 0	1	1	2	3	3	2	3	2	2	2	2	23/11 = 2.09
*m *= 1	2	2	*f*	5	*f*	3	5	4	*f*	3	3	27/8 = 3.375
*m *= 2	3	3	*f*	8	*f*	*f*	*f*	*f*	*f*	5	*f*	19/4 = 4.75
*m *= 3	4	4	*f*	*f*	*f*	*f*	*f*	*f*	*f*	*f*	*f*	8/2 = 4
*m *= 4	5	5	*f*	*f*	*f*	*f*	*f*	*f*	*f*	*f*	*f*	10/2 = 5
*m *= 5	6	*f*	*f*	*f*	*f*	*f*	*f*	*f*	*f*	*f*	*f*	6/1 = 6
*m *= 6	7	*f*	*f*	*f*	*f*	*f*	*f*	*f*	*f*	*f*	*f*	7/1 = 7

*f*: fail to contain enough SNPs for tolerating *m *missing SNPs

## Discussion

In terms of efficiency, the first and second greedy algorithms are faster than the LP-relaxation algorithm. The greedy algorithms usually returns a solution in seconds and the LP-relaxation algorithm requires about half minute for a solution. It is because the running time of LP-relaxation algorithm is bounded by the time of solving the LP problem. Furthermore, this LP-relaxation algorithm is repeated for 10 times to explore 10 different solutions. The OPT program for searching the optimal solution is apparently slower than the others. The optimal solution usually can not be found within a reasonable period of time if the size of the block becomes large. ¿From our empirical study, the optimal solution can be found in reasonable time by the OPT program if the block contains less than 20 SNPs (e.g., the short random data sets). But for those large data sets with more than 40 SNPs, the OPT program is significantly outperformed by the approximation algorithms (e.g., fail to output a solution within one week computation).

Assuming no missing data (i.e., *m *= 0), we compare the solutions found by each algorithm with the optimal solution. Table 2 lists the numbers of total tag SNPs found by each algorithm in previous experiments. In the experiments on random and Daly's data, the solution found by each algorithm is as good as the optimal solution. In the experiments on Hudson's and Patil's data, these algorithms still find solutions quite close to the optimal solution. For example, the approximation ratios of these algorithms are only 472443≈1.07
 MathType@MTEF@5@5@+=feaafiart1ev1aaatCvAUfKttLearuWrP9MDH5MBPbIqV92AaeXatLxBI9gBaebbnrfifHhDYfgasaacH8akY=wiFfYdH8Gipec8Eeeu0xXdbba9frFj0=OqFfea0dXdd9vqai=hGuQ8kuc9pgc9s8qqaq=dirpe0xb9q8qiLsFr0=vr0=vr0dc8meaabaqaciGacaGaaeqabaqabeGadaaakeaadaWcaaqaaiabisda0iabiEda3iabikdaYaqaaiabisda0iabisda0iabiodaZaaacqGHijYUcqaIXaqmcqGGUaGlcqaIWaamcqaI3aWnaaa@37F1@ and 46574595≈1.01
 MathType@MTEF@5@5@+=feaafiart1ev1aaatCvAUfKttLearuWrP9MDH5MBPbIqV92AaeXatLxBI9gBaebbnrfifHhDYfgasaacH8akY=wiFfYdH8Gipec8Eeeu0xXdbba9frFj0=OqFfea0dXdd9vqai=hGuQ8kuc9pgc9s8qqaq=dirpe0xb9q8qiLsFr0=vr0=vr0dc8meaabaqaciGacaGaaeqabaqabeGadaaakeaadaWcaaqaaiabisda0iabiAda2iabiwda1iabiEda3aqaaiabisda0iabiwda1iabiMda5iabiwda1aaacqGHijYUcqaIXaqmcqGGUaGlcqaIWaamcqaIXaqmaaa@39EB@, respectively.

**Table 2:** The number of total tag SNPs found by each algorithm. The percentage of tag SNPs with respect to total SNPs is shown in parentheses.

	Random data		Hudson's data		Patil's data	Daly's data
Total blocks	100	100	100	100	4135	11
Total SNPs	2000	4000	2000	4000	24047	103

1^*st *^Greedy	400 (20%)	400 (10%)	509 (25.5%)	472 (11.8%)	4610 (19.2%)	23 (22.3%)
2^*nd *^Greedy	400 (20%)	400 (10%)	509 (25.5%)	472 (11.8%)	4610 (19.2%)	23 (22.3%)
LP-relaxation	400 (20%)	400 (10%)	509 (25.5%)	471 (11.8%)	4657 (19.4%)	23 (22.3%)
OPT	400 (20%)	400 (10%)	492 (24.6%)	443 (11.1%)	4595 (19.1%)	23 (22.3%)

We then analyze the genotyping cost that can be saved by using tag SNPs. In Table 2, the percentage of tag SNPs in each data set is shown in parentheses. The experimental results indicate that the cost of genotyping tag SNPs is significantly reduced in comparison with genotyping all SNPs in a block. For example, in Patil's data, we only need to genotype about 19% of tag SNPs in each block, which saves about 81% genotyping cost.

The tradeoffs between the number of additional tag SNPs required and the number of missing SNPs allowed are discussed in the following. In practice, missing data in the genotyping experiment are usually limited to certain missing rate. We transform the maximum number of missing SNPs allowed into maximum missing rates allowed by calculating the percentage of *m *with respect to the number of robust tag SNPs. Table 3 lists the results of the first greedy algorithm applied on random and Hudson's long data sets. The number of additional tag SNPs grows with respect to *m *linearly. However, we observe that the maximum missing rate allowed grows slowly as *m *becomes large. This is because more additional tag SNPs are required in order to tolerate more missing SNPs. But under the same SNP missing rate, genotyping these additional tag SNPs may also increase the number of missing SNPs, which reduces the power of robust tag SNPs. On the positive side, when *m *is small, the corresponding maximum missing rate allowed is sufficient for most genotyping experiments since their missing rates are usually less than 10%. For example, the robust tag SNPs with *m *= 1 are sufficient to tolerate 10% missing SNPs, and they only requires at most 3 additional SNPs. As a result, genotyping additional tag SNPs for tolerating missing data is cost-effective under the current genotyping environment.

**Table 3:** The tradeoffs between additional tag SNPs required and maximum missing rates allowed. These results come from the first greedy algorithm applied on random and Hudson's data sets with 40 SNPs.

	*m*	0	1	2	3	4	5
Random data (40 SNPs)	average number of robust tag SNPs	4	6	8.51	10.47	12.89	14.92
	corresponding SNP missing rate	0	16.7%	23.5%	28.6%	31.0%	33.5%
	average number of extra tag SNPs	0	2	4.51	6.47	8.89	10.92

Hudson's data (40 SNPs)	average number of robust tag SNPs	4.72	7.71	11.28	14.67	18.23	21.67
	corresponding SNP missing rate	0	13.0%	17.7%	20.4%	21.9%	23.1%
	average number of extra tag SNPs	0	2.99	6.56	9.95	13.51	16.95

In reality, not all haplotypes are of equal importance or confidence. When selecting robust tag SNPs, it might be desirable to weight them according to their population frequency. To incorporate the frequency of haplotypes into this problem, there are two possible ways:

1. It can be easily done by discarding the rare haplotypes and retain the common haplotypes as the input of our algorithms. This approach would not require modification to our algorithms. But the retained common haplotypes will be processed as equally weighted.

2. Our algorithms try to find a set of SNPs such that each pair of haplotypes are distinguished by a threshold of at least (*m *+ 1) SNPs. A simplest way to weight the haplotypes is choosing different thresholds for each pair of haplotypes according to their population frequency. The haplotype pairs with higher frequency can then be assigned with more tag SNPs than the lower ones by our algorithms.

## Conclusion

In this paper, we show there exists a set of robust tag SNPs which is able to tolerate a number of missing data. Our study indicates that genotyping robust tag SNPs is more practical than genotyping minimum tag SNPs for association studies if we can not avoid the occurrence of missing data. We describe two greedy and one LP-relaxation approximation algorithms for finding robust tag SNPs. Our experimental results and theoretical analysis show that these algorithms are not only efficient but the solutions found are also close to the optimal solution. In terms of genotyping cost, we observe that the genotyping cost saved by using robust tag SNPs is significant, and genotyping additional tag SNPs to tolerate missing data is still cost-effective. One future direction is to assign weights to different types of SNPs (e.g., SNPs in coding or non-coding regions), and design algorithms for the selection of weighted tag SNPs.

## Software availability

**Project name: **efficient algorithms for utilizing SNP information.

**Project home page: **

**Operating system: **the implemented greedy algorithms are platform independent, and the implemented LP-relaxation algorithm runs on the Windows operating system.

**Programming language: **the greedy algorithms are implemented in JAVA, and the LP-relaxation algorithm is implemented in Perl.

## Methods

**Figure 6:**
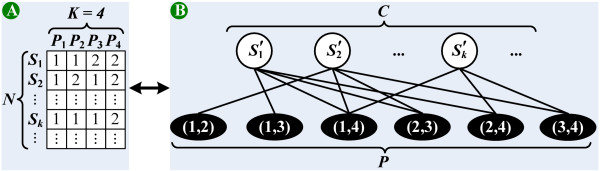
**Reformulation of the MRTS problem**. (A) The haplotype matrix *M*_*h *_containing *N *SNPs and *K *haplotype patterns. (B) The bipartite graph corresponding to *M*_*h*_.

Assume we are given a haplotype block containing *N *SNPs and *K *haplotype patterns. This block is denoted by an *N *× *K *binary matrix *M*_*h *_(see Figure 6 (A)). Define *M*_*h*_[*i*,*j*] ∈ {1,2} for each *i *∈ [1, *N*] and *j *∈ [1, *K*], where 1 and 2 represent the major and minor alleles, respectively. In reality, the haplotype block may also contain missing data. This formulation can be easily extended to handle missing data by treating them as wild card symbols. To simplify the presentation of this paper, we will assume no missing data in the block. Let *C *be the set of all SNPs in *M*_*h*_. The robust tag SNPs *C' *⊆ *C *are a subset of SNPs which is able to distinguish each pair of haplotype patterns unambiguously when at most *m *SNPs are missing. Note that the missing data may occur at any SNP locus and thus create different missing patterns (see Figure 2). For any haplotype pattern with up to *m *missing SNPs, the set of robust tag SNPs *C' *is required to distinguish all of them unambiguously.

To distinguish a haplotype pattern unambiguously, each pair of patterns must be distinguished by at least one SNP in *C'*. For example (see Figure 6 (A)), we say patterns *P*_1 _and *P*_2 _can be distinguished by SNP *S*_2 _since *M*_*h*_[2,1] ≠ *M*_*h*_[2,2]. A formal definition of this problem is given below.

### Problem: Minimum Robust Tag SNPs (MRTS)

**Input: **An *N *× *K *matrix *M*_*h *_and an integer *m*.

**Output: **The minimum subset of SNPs *C' *⊆ *C *which satisfies:

(1) for each pair of patterns *P*_*i *_and *P*_*j*_, these is a SNP *S*_*k *_∈ *C' *such that *M*_*h*_[*k*, *i*] ≠ *M*_*h*_[*k*, *j*];

(2) when at most *m *SNPs are discarded from *C' *arbitrarily, (1) still holds.

We then reformulate MRTS to a variant of the *set covering problem *[6]. Each SNP *S*_*k *_∈ *C *(i.e., the *k*-th row in *M*_*h*_) is reformulated to a set Sk'
 MathType@MTEF@5@5@+=feaafiart1ev1aaatCvAUfKttLearuWrP9MDH5MBPbIqV92AaeXatLxBI9gBaebbnrfifHhDYfgasaacH8akY=wiFfYdH8Gipec8Eeeu0xXdbba9frFj0=OqFfea0dXdd9vqai=hGuQ8kuc9pgc9s8qqaq=dirpe0xb9q8qiLsFr0=vr0=vr0dc8meaabaqaciGacaGaaeqabaqabeGadaaakeaacqWGtbWudaqhaaWcbaGaem4AaSgabaGaei4jaCcaaaaa@303F@ = {(*i*, *j*) | *M*[*k*, *i*] ≠ *M*[*k*, *j*] and *i *<*j*}. For example, suppose the *k*-th row in *M*_*h *_is {1,1,1,2}. The corresponding set Sk'
 MathType@MTEF@5@5@+=feaafiart1ev1aaatCvAUfKttLearuWrP9MDH5MBPbIqV92AaeXatLxBI9gBaebbnrfifHhDYfgasaacH8akY=wiFfYdH8Gipec8Eeeu0xXdbba9frFj0=OqFfea0dXdd9vqai=hGuQ8kuc9pgc9s8qqaq=dirpe0xb9q8qiLsFr0=vr0=vr0dc8meaabaqaciGacaGaaeqabaqabeGadaaakeaacqWGtbWudaqhaaWcbaGaem4AaSgabaGaei4jaCcaaaaa@303F@ = {(1,4), (2,4), (3,4)}. In other words, Sk'
 MathType@MTEF@5@5@+=feaafiart1ev1aaatCvAUfKttLearuWrP9MDH5MBPbIqV92AaeXatLxBI9gBaebbnrfifHhDYfgasaacH8akY=wiFfYdH8Gipec8Eeeu0xXdbba9frFj0=OqFfea0dXdd9vqai=hGuQ8kuc9pgc9s8qqaq=dirpe0xb9q8qiLsFr0=vr0=vr0dc8meaabaqaciGacaGaaeqabaqabeGadaaakeaacqWGtbWudaqhaaWcbaGaem4AaSgabaGaei4jaCcaaaaa@303F@ stores the pairs of patterns distinguished by SNP *S*_*k*_. Define *P *as the set that contains all pairs of patterns (i.e., *P *= {(*i*,*j*) | 1 ≤ *i *<*j *≤ *K*} = {(1,2), (1,3), ..., (*K *- l,*K*)}).

Consider each element in *P *and each reformulated set of *C *as nodes in an undirected bipartite graph (see Figure 6 (B). If SNP *S*_*k *_can distinguish patterns *P*_*i *_and *P*_*j *_(i.e., (*i*,*j*) ∈ Sk'
 MathType@MTEF@5@5@+=feaafiart1ev1aaatCvAUfKttLearuWrP9MDH5MBPbIqV92AaeXatLxBI9gBaebbnrfifHhDYfgasaacH8akY=wiFfYdH8Gipec8Eeeu0xXdbba9frFj0=OqFfea0dXdd9vqai=hGuQ8kuc9pgc9s8qqaq=dirpe0xb9q8qiLsFr0=vr0=vr0dc8meaabaqaciGacaGaaeqabaqabeGadaaakeaacqWGtbWudaqhaaWcbaGaem4AaSgabaGaei4jaCcaaaaa@303F@), there is an edge connecting the nodes (*i*, *j*) and Sk'
 MathType@MTEF@5@5@+=feaafiart1ev1aaatCvAUfKttLearuWrP9MDH5MBPbIqV92AaeXatLxBI9gBaebbnrfifHhDYfgasaacH8akY=wiFfYdH8Gipec8Eeeu0xXdbba9frFj0=OqFfea0dXdd9vqai=hGuQ8kuc9pgc9s8qqaq=dirpe0xb9q8qiLsFr0=vr0=vr0dc8meaabaqaciGacaGaaeqabaqabeGadaaakeaacqWGtbWudaqhaaWcbaGaem4AaSgabaGaei4jaCcaaaaa@303F@. The following lemma implies that each pair of patterns must be distinguished by at least (*m *+ 1) SNPs to tolerate *m *missing SNPs.

**Lemma 1. ***C' *⊆ *C is the set of robust tag SNPs which allows at most m missing SNPs iff each node in P has at least *(*m *+ 1) *edges connecting to each node in C'*.

*Proof. *Let *C' *be the set of robust tag SNPs which allows at most *m *missing SNPs. Suppose patterns *P*_*i *_and *P*_*j *_are distinguished by only *m *SNPs in *C' *(i.e., (*i*, *j*) has only *m *edges connecting to nodes in *C'*). However, if these *m *SNPs are all missing, no other SNPs in *C' *are able to distinguish patterns *P*_*i *_and *P*_*j*_, which is a contradiction. Thus, each pair of patterns must be distinguished by at least (*m *+ 1) SNPs, which implies that each node in *P *must have at least (*m *+ 1) edges connecting to nodes in *C'*. The proof of the other direction is similar.   

In the following, we give a lower bound regarding the minimum number of robust tag SNPs required, which is used to skip some solution space by the OPT program.

**Lemma 2. ***Given K haplotype patterns, the minimum number of robust tag SNPs required is at least *log *K*.

*Proof. *Recall that the value of a SNP is binary. The maximum number of distinct haplotypes which can be distinguished by *N *SNPs is at most 2^*N*^. As a result, for a given data set containing *K *haplotype patterns, the minimum number of SNPs required is at least log *K*.    

The following theorem shows the NP-hardness of the MRTS problem, which implies there is no polynomial time algorithm to find the optimal solution of MRTS.

**Theorem 1. ***The MRTS problem is NP-hard*.

*Proof. *When *m *= 0, MRTS is the same as the original problem of finding minimum tag SNPs, which is known as the *minimum test set *problem [6,17]. Since the minimum test set problem is NP-hard and can be reduced to a special case of MRTS, MRTS is NP-hard.    

### The first greedy algorithm

**Figure 7:**
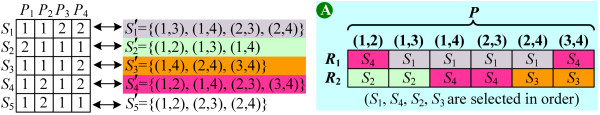
**An example of the first greedy algorithm**. The SNPs *S*_1_, *S*_4_, *S*_2_, and *S*_3 _are selected by the first greedy algorithm. (A) The table that stores each selected SNP.

To solve MRTS efficiently, we propose a greedy algorithm which returns a solution not too larger than the optimal solution. By Lemma 1, to tolerate *m *missing tag SNPs, we need to find a subset of SNPs *C' *⊆ *C *such that each pair of patterns in *P *is distinguished by at least (*m + *1) SNPs in *C'*. Assume that the SNPs selected by this algorithm are stored in a (*m *+ 1) × |*P*| table (see Figure 7 (A)). Initially, each grid in the table is empty. Once a SNP *S*_*k*_, (that can distinguish patterns *P*_*i *_and *P*_*j*_) is selected, one grid of the column (*i*, *j*) is filled in with *S*_*k*_, and we say that this grid is *covered *by *S*_*k*_.

This greedy algorithm works by covering the grids from the first row to the (*m *+ 1)-th row, and greedily selects a SNP which covers most uncovered grids in the *i*-th row at each iteration. In other words, while working on the *i*-th row, a SNP is selected if its reformulated set *S' *maximizes |*S' *∩ *R*_*i *_|, where *R*_*i *_is the set of uncovered grids at the *i*-th row.

Figure 7 illustrates an example for this algorithm to tolerate one missing tag SNP (i.e., *m *= 1). The SNPs *S*_1_, *S*_4_, *S*_2_, and *S*_3 _are selected in order. When all grids in this table are covered, each pair of patterns is distinguished by (*m + *1) SNPs in the corresponding column. Thus, the SNPs in this table are the robust tag SNPs which can tolerate up to *m *missing SNPs. The pseudo code of this greedy algorithm is given below.

**Algorithm: **FlRST-GREEDY-ALGORITHM (*C*, *P*, *m*)

1 *R*_*i *_← *P*, ∀*i *∈ [1, *m *+ 1]

2 *C' *← *φ*

3 **for ***i *= 1 to *m *+ 1 **do**

4    **while ***R*_*i *_≠ *φ ***do**

5       select and remove a SNP *S *from *C *that maximizes |*S' *∩ *R*_*i*_|

6       *C' *← *C' *∪ *S*

7       *j *← *i*

8       **while ***S' *≠ *φ ***and ***j *≤ *m *+ 1 **do**

9          *S*_*tmp *_← *S' *∩ *R*_*j *_//*S*_*tmp *_is a temporary variable for holding the result of *S' *∩ *R*_*i*_

10          *R*_*j *_← *R*_*j *_- *S*_*tmp*_

11          *S' *← *S' *- *S*_*tmp*_

12          *j *← *j *+ l

13       **endwhile**

14    **endwhile**

15 **endfor**

16 **return ***C'*

The time complexity of this algorithm is analyzed as follows. At Line 4, the number of iterations of the intermediate loop is bounded by |*R*_*i*_| ≤ |*P*|. Within the loop body (Lines 5–13), Line 5 takes *O*(|*C*||*P*|) because we need to check all SNPs in *C *and examine the uncovered grids of *R*_*i*_. The inner loop (Lines 8–13) takes only *O*(|*S'*|). Thus, the entire program runs in *O*(*m*|*C*||*P*|^2^).

**Figure 8:**
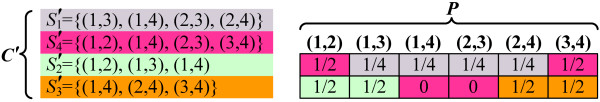
**Analysis of the first greedy algorithm**. This figure shows the cost Cji
 MathType@MTEF@5@5@+=feaafiart1ev1aaatCvAUfKttLearuWrP9MDH5MBPbIqV92AaeXatLxBI9gBaebbnrfifHhDYfgasaacH8akY=wiFfYdH8Gipec8Eeeu0xXdbba9frFj0=OqFfea0dXdd9vqai=hGuQ8kuc9pgc9s8qqaq=dirpe0xb9q8qiLsFr0=vr0=vr0dc8meaabaqaciGacaGaaeqabaqabeGadaaakeaacqWGdbWqdaqhaaWcbaGaemOAaOgabaGaemyAaKgaaaaa@30A2@ of each grid for the first greedy algorithm.

We now show the solution *C' *returned by the first greedy algorithm is not too larger than the optimal solution C*. Suppose the algorithm selects the *k*-th SNP when working on the *i*-th row. Let |Skc
 MathType@MTEF@5@5@+=feaafiart1ev1aaatCvAUfKttLearuWrP9MDH5MBPbIqV92AaeXatLxBI9gBaebbnrfifHhDYfgasaacH8akY=wiFfYdH8Gipec8Eeeu0xXdbba9frFj0=OqFfea0dXdd9vqai=hGuQ8kuc9pgc9s8qqaq=dirpe0xb9q8qiLsFr0=vr0=vr0dc8meaabaqaciGacaGaaeqabaqabeGadaaakeaacqWGtbWudaqhaaWcbaGaem4AaSgabaGaem4yamgaaaaa@30B8@| be the number of grids in the *i*-th row covered by the *k*-th selected SNP (i.e., |Skc
 MathType@MTEF@5@5@+=feaafiart1ev1aaatCvAUfKttLearuWrP9MDH5MBPbIqV92AaeXatLxBI9gBaebbnrfifHhDYfgasaacH8akY=wiFfYdH8Gipec8Eeeu0xXdbba9frFj0=OqFfea0dXdd9vqai=hGuQ8kuc9pgc9s8qqaq=dirpe0xb9q8qiLsFr0=vr0=vr0dc8meaabaqaciGacaGaaeqabaqabeGadaaakeaacqWGtbWudaqhaaWcbaGaem4AaSgabaGaem4yamgaaaaa@30B8@| = |*S*' ∩ *R*_*i*_|; see Line 5 in FIRST-GREEDY-ALGORITHM). For example (see Figure 7), S2c=2
 MathType@MTEF@5@5@+=feaafiart1ev1aaatCvAUfKttLearuWrP9MDH5MBPbIqV92AaeXatLxBI9gBaebbnrfifHhDYfgasaacH8akY=wiFfYdH8Gipec8Eeeu0xXdbba9frFj0=OqFfea0dXdd9vqai=hGuQ8kuc9pgc9s8qqaq=dirpe0xb9q8qiLsFr0=vr0=vr0dc8meaabaqaciGacaGaaeqabaqabeGadaaakeaacqWGtbWudaqhaaWcbaGaeGOmaidabaGaem4yamgaaOGaeyypa0JaeGOmaidaaa@324D@ since the second selected SNP (i.e., *S*_4_) covers two grids in the first row. We incur 1 unit of cost to each selected SNP, and spread this cost among the grids in Skc
 MathType@MTEF@5@5@+=feaafiart1ev1aaatCvAUfKttLearuWrP9MDH5MBPbIqV92AaeXatLxBI9gBaebbnrfifHhDYfgasaacH8akY=wiFfYdH8Gipec8Eeeu0xXdbba9frFj0=OqFfea0dXdd9vqai=hGuQ8kuc9pgc9s8qqaq=dirpe0xb9q8qiLsFr0=vr0=vr0dc8meaabaqaciGacaGaaeqabaqabeGadaaakeaacqWGtbWudaqhaaWcbaGaem4AaSgabaGaem4yamgaaaaa@30B8@[3]. In other words, each grid at the *i-*th row and *j*-th column is assigned a cost Cji
 MathType@MTEF@5@5@+=feaafiart1ev1aaatCvAUfKttLearuWrP9MDH5MBPbIqV92AaeXatLxBI9gBaebbnrfifHhDYfgasaacH8akY=wiFfYdH8Gipec8Eeeu0xXdbba9frFj0=OqFfea0dXdd9vqai=hGuQ8kuc9pgc9s8qqaq=dirpe0xb9q8qiLsFr0=vr0=vr0dc8meaabaqaciGacaGaaeqabaqabeGadaaakeaacqWGdbWqdaqhaaWcbaGaemOAaOgabaGaemyAaKgaaaaa@30A2@ (see Figure 8), where

Cji={1|Skc|if the algorithm selects the k-th SNP when covering the i-th row;  0otherwise.
 MathType@MTEF@5@5@+=feaafiart1ev1aaatCvAUfKttLearuWrP9MDH5MBPbIqV92AaeXatLxBI9gBaebbnrfifHhDYfgasaacH8akY=wiFfYdH8Gipec8Eeeu0xXdbba9frFj0=OqFfea0dXdd9vqai=hGuQ8kuc9pgc9s8qqaq=dirpe0xb9q8qiLsFr0=vr0=vr0dc8meaabaqaciGacaGaaeqabaqabeGadaaakeaacqWGdbWqdaqhaaWcbaGaemOAaOgabaGaemyAaKgaaOGaeyypa0ZaaiqaaeaafaqaaeGacaaabaWaaSqaaSqaaiabigdaXaqaamaaemaabaGaem4uam1aa0baaWqaaiabdUgaRbqaaiabdogaJbaaaSGaay5bSlaawIa7aaaaaOqaaiabbMgaPjabbAgaMjabbccaGiabbsha0jabbIgaOjabbwgaLjabbccaGiabbggaHjabbYgaSjabbEgaNjabb+gaVjabbkhaYjabbMgaPjabbsha0jabbIgaOjabb2gaTjabbccaGiabbohaZjabbwgaLjabbYgaSjabbwgaLjabbogaJjabbsha0jabbohaZjabbccaGiabbsha0jabbIgaOjabbwgaLjabbccaGiabdUgaRjabb2caTiabbsha0jabbIgaOjabbccaGiabbofatjabb6eaojabbcfaqjabbccaGiabbEha3jabbIgaOjabbwgaLjabb6gaUjabbccaGiabbogaJjabb+gaVjabbAha2jabbwgaLjabbkhaYjabbMgaPjabb6gaUjabbEgaNjabbccaGiabbsha0jabbIgaOjabbwgaLjabbccaGiabdMgaPjabb2caTiabbsha0jabbIgaOjabbccaGiabbkhaYjabb+gaVjabbEha3jabbUda7aqaaiaaykW7caaMc8UaeGimaadabaGaee4Ba8MaeeiDaqNaeeiAaGMaeeyzauMaeeOCaiNaee4DaCNaeeyAaKMaee4CamNaeeyzauMaeeOla4caaaGaay5Eaaaaaa@9D18@

Since each selected SNP is assigned 1 unit of cost, the sum of Cji
 MathType@MTEF@5@5@+=feaafiart1ev1aaatCvAUfKttLearuWrP9MDH5MBPbIqV92AaeXatLxBI9gBaebbnrfifHhDYfgasaacH8akY=wiFfYdH8Gipec8Eeeu0xXdbba9frFj0=OqFfea0dXdd9vqai=hGuQ8kuc9pgc9s8qqaq=dirpe0xb9q8qiLsFr0=vr0=vr0dc8meaabaqaciGacaGaaeqabaqabeGadaaakeaacqWGdbWqdaqhaaWcbaGaemOAaOgabaGaemyAaKgaaaaa@30A2@ for each grid in the table is equal to |*C'*|,

i.e.,

|C'|  =∑i=1m+1∑j=1K(K−1)2Cji.     (1)
 MathType@MTEF@5@5@+=feaafiart1ev1aaatCvAUfKttLearuWrP9MDH5MBPbIqV92AaeXatLxBI9gBaebbnrfifHhDYfgasaacH8akY=wiFfYdH8Gipec8Eeeu0xXdbba9frFj0=OqFfea0dXdd9vqai=hGuQ8kuc9pgc9s8qqaq=dirpe0xb9q8qiLsFr0=vr0=vr0dc8meaabaqaciGacaGaaeqabaqabeGadaaakeaacqGG8baFcqWGdbWqcqGGNaWjcqGG8baFcaaMc8UaaGPaVlabg2da9maaqahabaWaaabCaeaacqWGdbWqdaqhaaWcbaGaemOAaOgabaGaemyAaKgaaaqaaiabdQgaQjabg2da9iabigdaXaqaamaaleaameaacqWGlbWscqGGOaakcqWGlbWscqGHsislcqaIXaqmcqGGPaqkaeaacqaIYaGmaaaaniabggHiLdaaleaacqWGPbqAcqGH9aqpcqaIXaqmaeaacqWGTbqBcqGHRaWkcqaIXaqma0GaeyyeIuoakiabc6caUiaaxMaacaWLjaWaaeWaaeaacqaIXaqmaiaawIcacaGLPaaaaaa@537C@

Let Rki
 MathType@MTEF@5@5@+=feaafiart1ev1aaatCvAUfKttLearuWrP9MDH5MBPbIqV92AaeXatLxBI9gBaebbnrfifHhDYfgasaacH8akY=wiFfYdH8Gipec8Eeeu0xXdbba9frFj0=OqFfea0dXdd9vqai=hGuQ8kuc9pgc9s8qqaq=dirpe0xb9q8qiLsFr0=vr0=vr0dc8meaabaqaciGacaGaaeqabaqabeGadaaakeaacqWGsbGudaqhaaWcbaGaem4AaSgabaGaemyAaKgaaaaa@30C2@ be the number of uncovered grids in the *i*-th row before the *k*-th iteration (i.e., (*k *- 1) SNPs have been selected by the algorithm). For example (see Figure 8), R21=2
 MathType@MTEF@5@5@+=feaafiart1ev1aaatCvAUfKttLearuWrP9MDH5MBPbIqV92AaeXatLxBI9gBaebbnrfifHhDYfgasaacH8akY=wiFfYdH8Gipec8Eeeu0xXdbba9frFj0=OqFfea0dXdd9vqai=hGuQ8kuc9pgc9s8qqaq=dirpe0xb9q8qiLsFr0=vr0=vr0dc8meaabaqaciGacaGaaeqabaqabeGadaaakeaacqWGsbGudaqhaaWcbaGaeGOmaidabaGaeGymaedaaOGaeyypa0JaeGOmaidaaa@31EC@ since two grids in the first row are still uncovered before the second SNP is selected. Define Ci'
 MathType@MTEF@5@5@+=feaafiart1ev1aaatCvAUfKttLearuWrP9MDH5MBPbIqV92AaeXatLxBI9gBaebbnrfifHhDYfgasaacH8akY=wiFfYdH8Gipec8Eeeu0xXdbba9frFj0=OqFfea0dXdd9vqai=hGuQ8kuc9pgc9s8qqaq=dirpe0xb9q8qiLsFr0=vr0=vr0dc8meaabaqaciGacaGaaeqabaqabeGadaaakeaacqWGdbWqdaqhaaWcbaGaemyAaKgabaGaei4jaCcaaaaa@301B@ as the set of iterations used by the algorithm when working on the *i*-th row. For example (see Figure 8), C2'={3,4}
 MathType@MTEF@5@5@+=feaafiart1ev1aaatCvAUfKttLearuWrP9MDH5MBPbIqV92AaeXatLxBI9gBaebbnrfifHhDYfgasaacH8akY=wiFfYdH8Gipec8Eeeu0xXdbba9frFj0=OqFfea0dXdd9vqai=hGuQ8kuc9pgc9s8qqaq=dirpe0xb9q8qiLsFr0=vr0=vr0dc8meaabaqaciGacaGaaeqabaqabeGadaaakeaacqWGdbWqdaqhaaWcbaGaeGOmaidabaGaei4jaCcaaOGaeyypa0Jaei4EaSNaeG4mamJaeiilaWIaeGinaqJaeiyFa0haaa@368C@ since this algorithm works on the second row in the third and fourth iterations. We can rewrite (1) as

∑i=1m+1∑j=1K(K−1)2Cji=∑i=1m+1∑k∈Ci'(Rk−1i−Rki)1|Skc|.     (2)
 MathType@MTEF@5@5@+=feaafiart1ev1aaatCvAUfKttLearuWrP9MDH5MBPbIqV92AaeXatLxBI9gBaebbnrfifHhDYfgasaacH8akY=wiFfYdH8Gipec8Eeeu0xXdbba9frFj0=OqFfea0dXdd9vqai=hGuQ8kuc9pgc9s8qqaq=dirpe0xb9q8qiLsFr0=vr0=vr0dc8meaabaqaciGacaGaaeqabaqabeGadaaakeaadaaeWbqaamaaqahabaGaem4qam0aa0baaSqaaiabdQgaQbqaaiabdMgaPbaaaeaacqWGQbGAcqGH9aqpcqaIXaqmaeaadaWcbaadbaGaem4saSKaeiikaGIaem4saSKaeyOeI0IaeGymaeJaeiykaKcabaGaeGOmaidaaaqdcqGHris5aaWcbaGaemyAaKMaeyypa0JaeGymaedabaGaemyBa0Maey4kaSIaeGymaedaniabggHiLdGccqGH9aqpdaaeWbqaamaaqafabaWaaeWaaeaacqWGsbGudaqhaaWcbaGaem4AaSMaeyOeI0IaeGymaedabaGaemyAaKgaaOGaeyOeI0IaemOuai1aa0baaSqaaiabdUgaRbqaaiabdMgaPbaaaOGaayjkaiaawMcaaaWcbaGaem4AaSMaeyicI4Saem4qam0aa0baaWqaaiabdMgaPbqaaiabcEcaNaaaaSqab0GaeyyeIuoaaSqaaiabdMgaPjabg2da9iabigdaXaqaaiabd2gaTjabgUcaRiabigdaXaqdcqGHris5aOWaaSaaaeaacqaIXaqmaeaacqGG8baFcqWGtbWudaqhaaWcbaGaem4AaSgabaGaem4yamgaaOGaeiiFaWhaaiabc6caUiaaxMaacaWLjaWaaeWaaeaacqaIYaGmaiaawIcacaGLPaaaaaa@7177@

**Lemma 3. ***The k-th selected SNP has *|Skc|≥Rk−1i|C*|
 MathType@MTEF@5@5@+=feaafiart1ev1aaatCvAUfKttLearuWrP9MDH5MBPbIqV92AaeXatLxBI9gBaebbnrfifHhDYfgasaacH8akY=wiFfYdH8Gipec8Eeeu0xXdbba9frFj0=OqFfea0dXdd9vqai=hGuQ8kuc9pgc9s8qqaq=dirpe0xb9q8qiLsFr0=vr0=vr0dc8meaabaqaciGacaGaaeqabaqabeGadaaakeaadaabdaqaaiabdofatnaaDaaaleaacqWGRbWAaeaacqWGJbWyaaaakiaawEa7caGLiWoacqGHLjYSdaWcaaqaaiabdkfasnaaDaaaleaacqWGRbWAcqGHsislcqaIXaqmaeaacqWGPbqAaaaakeaacqGG8baFcqWGdbWqcqGGQaGkcqGG8baFaaaaaa@40A0@.

*Proof. *Suppose the algorithm is working on the *i*-th row at the beginning of the *k*-th iteration. Let Ck*
 MathType@MTEF@5@5@+=feaafiart1ev1aaatCvAUfKttLearuWrP9MDH5MBPbIqV92AaeXatLxBI9gBaebbnrfifHhDYfgasaacH8akY=wiFfYdH8Gipec8Eeeu0xXdbba9frFj0=OqFfea0dXdd9vqai=hGuQ8kuc9pgc9s8qqaq=dirpe0xb9q8qiLsFr0=vr0=vr0dc8meaabaqaciGacaGaaeqabaqabeGadaaakeaacqWGdbWqdaqhaaWcbaGaem4AaSgabaGaeiOkaOcaaaaa@3025@ be the set of SNPs in C* (the optimal solution) that has been selected by the algorithm before the *k*-th iteration, and the set of remaining SNPs in C* be Ck¯*
 MathType@MTEF@5@5@+=feaafiart1ev1aaatCvAUfKttLearuWrP9MDH5MBPbIqV92AaeXatLxBI9gBaebbnrfifHhDYfgasaacH8akY=wiFfYdH8Gipec8Eeeu0xXdbba9frFj0=OqFfea0dXdd9vqai=hGuQ8kuc9pgc9s8qqaq=dirpe0xb9q8qiLsFr0=vr0=vr0dc8meaabaqaciGacaGaaeqabaqabeGadaaakeaacqWGdbWqdaqhaaWcbaGafm4AaSMbaebaaeaacqGGQaGkaaaaaa@303D@. We claim that there exists a SNP in Ck¯*
 MathType@MTEF@5@5@+=feaafiart1ev1aaatCvAUfKttLearuWrP9MDH5MBPbIqV92AaeXatLxBI9gBaebbnrfifHhDYfgasaacH8akY=wiFfYdH8Gipec8Eeeu0xXdbba9frFj0=OqFfea0dXdd9vqai=hGuQ8kuc9pgc9s8qqaq=dirpe0xb9q8qiLsFr0=vr0=vr0dc8meaabaqaciGacaGaaeqabaqabeGadaaakeaacqWGdbWqdaqhaaWcbaGafm4AaSMbaebaaeaacqGGQaGkaaaaaa@303D@ which can cover at least Rki|Ck¯*|
 MathType@MTEF@5@5@+=feaafiart1ev1aaatCvAUfKttLearuWrP9MDH5MBPbIqV92AaeXatLxBI9gBaebbnrfifHhDYfgasaacH8akY=wiFfYdH8Gipec8Eeeu0xXdbba9frFj0=OqFfea0dXdd9vqai=hGuQ8kuc9pgc9s8qqaq=dirpe0xb9q8qiLsFr0=vr0=vr0dc8meaabaqaciGacaGaaeqabaqabeGadaaakeaadaWcaaqaaiabdkfasnaaDaaaleaacqWGRbWAaeaacqWGPbqAaaaakeaadaabdaqaaiabdoeadnaaDaaaleaacuWGRbWAgaqeaaqaaiabcQcaQaaaaOGaay5bSlaawIa7aaaaaaa@3797@ grids in the *i*-th row. Otherwise (i.e., each SNP in Ck¯*
 MathType@MTEF@5@5@+=feaafiart1ev1aaatCvAUfKttLearuWrP9MDH5MBPbIqV92AaeXatLxBI9gBaebbnrfifHhDYfgasaacH8akY=wiFfYdH8Gipec8Eeeu0xXdbba9frFj0=OqFfea0dXdd9vqai=hGuQ8kuc9pgc9s8qqaq=dirpe0xb9q8qiLsFr0=vr0=vr0dc8meaabaqaciGacaGaaeqabaqabeGadaaakeaacqWGdbWqdaqhaaWcbaGafm4AaSMbaebaaeaacqGGQaGkaaaaaa@303D@ covers less than Rki|Ck¯*|
 MathType@MTEF@5@5@+=feaafiart1ev1aaatCvAUfKttLearuWrP9MDH5MBPbIqV92AaeXatLxBI9gBaebbnrfifHhDYfgasaacH8akY=wiFfYdH8Gipec8Eeeu0xXdbba9frFj0=OqFfea0dXdd9vqai=hGuQ8kuc9pgc9s8qqaq=dirpe0xb9q8qiLsFr0=vr0=vr0dc8meaabaqaciGacaGaaeqabaqabeGadaaakeaadaWcaaqaaiabdkfasnaaDaaaleaacqWGRbWAaeaacqWGPbqAaaaakeaadaabdaqaaiabdoeadnaaDaaaleaacuWGRbWAgaqeaaqaaiabcQcaQaaaaOGaay5bSlaawIa7aaaaaaa@3797@ grids), all SNPs in Ck¯*
 MathType@MTEF@5@5@+=feaafiart1ev1aaatCvAUfKttLearuWrP9MDH5MBPbIqV92AaeXatLxBI9gBaebbnrfifHhDYfgasaacH8akY=wiFfYdH8Gipec8Eeeu0xXdbba9frFj0=OqFfea0dXdd9vqai=hGuQ8kuc9pgc9s8qqaq=dirpe0xb9q8qiLsFr0=vr0=vr0dc8meaabaqaciGacaGaaeqabaqabeGadaaakeaacqWGdbWqdaqhaaWcbaGafm4AaSMbaebaaeaacqGGQaGkaaaaaa@303D@ will cover less than (Rki|Ck¯*|×|Ck¯*|=Rki)
 MathType@MTEF@5@5@+=feaafiart1ev1aaatCvAUfKttLearuWrP9MDH5MBPbIqV92AaeXatLxBI9gBaebbnrfifHhDYfgasaacH8akY=wiFfYdH8Gipec8Eeeu0xXdbba9frFj0=OqFfea0dXdd9vqai=hGuQ8kuc9pgc9s8qqaq=dirpe0xb9q8qiLsFr0=vr0=vr0dc8meaabaqaciGacaGaaeqabaqabeGadaaakeaacqGGOaakdaWcaaqaaiabdkfasnaaDaaaleaacqWGRbWAaeaacqWGPbqAaaaakeaadaabdaqaaiabdoeadnaaDaaaleaacuWGRbWAgaqeaaqaaiabcQcaQaaaaOGaay5bSlaawIa7aaaacqGHxdaTdaabdaqaaiabdoeadnaaDaaaleaacuWGRbWAgaqeaaqaaiabcQcaQaaaaOGaay5bSlaawIa7aiabg2da9iabdkfasnaaDaaaleaacqWGRbWAaeaacqWGPbqAaaGccqGGPaqkaaa@473F@ grids in the *i-th *row. But since Ck*∪Ck¯*=C*
 MathType@MTEF@5@5@+=feaafiart1ev1aaatCvAUfKttLearuWrP9MDH5MBPbIqV92AaeXatLxBI9gBaebbnrfifHhDYfgasaacH8akY=wiFfYdH8Gipec8Eeeu0xXdbba9frFj0=OqFfea0dXdd9vqai=hGuQ8kuc9pgc9s8qqaq=dirpe0xb9q8qiLsFr0=vr0=vr0dc8meaabaqaciGacaGaaeqabaqabeGadaaakeaacqWGdbWqdaqhaaWcbaGaem4AaSgabaGaeiOkaOcaaOGaeSOkIuLaem4qam0aa0baaSqaaiqbdUgaRzaaraaabaGaeiOkaOcaaOGaeyypa0Jaem4qamKaeiOkaOcaaa@37EB@, this implies that C* can not cover all grids in Rki
 MathType@MTEF@5@5@+=feaafiart1ev1aaatCvAUfKttLearuWrP9MDH5MBPbIqV92AaeXatLxBI9gBaebbnrfifHhDYfgasaacH8akY=wiFfYdH8Gipec8Eeeu0xXdbba9frFj0=OqFfea0dXdd9vqai=hGuQ8kuc9pgc9s8qqaq=dirpe0xb9q8qiLsFr0=vr0=vr0dc8meaabaqaciGacaGaaeqabaqabeGadaaakeaacqWGsbGudaqhaaWcbaGaem4AaSgabaGaemyAaKgaaaaa@30C2@, which is a contradiction. Because all SNPs in Ck¯*
 MathType@MTEF@5@5@+=feaafiart1ev1aaatCvAUfKttLearuWrP9MDH5MBPbIqV92AaeXatLxBI9gBaebbnrfifHhDYfgasaacH8akY=wiFfYdH8Gipec8Eeeu0xXdbba9frFj0=OqFfea0dXdd9vqai=hGuQ8kuc9pgc9s8qqaq=dirpe0xb9q8qiLsFr0=vr0=vr0dc8meaabaqaciGacaGaaeqabaqabeGadaaakeaacqWGdbWqdaqhaaWcbaGafm4AaSMbaebaaeaacqGGQaGkaaaaaa@303D@ are candidates to the greedy algorithm, the *k*-th selected SNP must cover at least Rki|Ck¯*|
 MathType@MTEF@5@5@+=feaafiart1ev1aaatCvAUfKttLearuWrP9MDH5MBPbIqV92AaeXatLxBI9gBaebbnrfifHhDYfgasaacH8akY=wiFfYdH8Gipec8Eeeu0xXdbba9frFj0=OqFfea0dXdd9vqai=hGuQ8kuc9pgc9s8qqaq=dirpe0xb9q8qiLsFr0=vr0=vr0dc8meaabaqaciGacaGaaeqabaqabeGadaaakeaadaWcaaqaaiabdkfasnaaDaaaleaacqWGRbWAaeaacqWGPbqAaaaakeaadaabdaqaaiabdoeadnaaDaaaleaacuWGRbWAgaqeaaqaaiabcQcaQaaaaOGaay5bSlaawIa7aaaaaaa@3797@ grids in the *i*-th row, which implies |Skc|≥Rk−1i|C*|
 MathType@MTEF@5@5@+=feaafiart1ev1aaatCvAUfKttLearuWrP9MDH5MBPbIqV92AaeXatLxBI9gBaebbnrfifHhDYfgasaacH8akY=wiFfYdH8Gipec8Eeeu0xXdbba9frFj0=OqFfea0dXdd9vqai=hGuQ8kuc9pgc9s8qqaq=dirpe0xb9q8qiLsFr0=vr0=vr0dc8meaabaqaciGacaGaaeqabaqabeGadaaakeaadaabdaqaaiabdofatnaaDaaaleaacqWGRbWAaeaacqWGJbWyaaaakiaawEa7caGLiWoacqGHLjYSdaWcaaqaaiabdkfasnaaDaaaleaacqWGRbWAcqGHsislcqaIXaqmaeaacqWGPbqAaaaakeaacqGG8baFcqWGdbWqcqGGQaGkcqGG8baFaaaaaa@40A0@ since |C*| ≥ |Ck¯*
 MathType@MTEF@5@5@+=feaafiart1ev1aaatCvAUfKttLearuWrP9MDH5MBPbIqV92AaeXatLxBI9gBaebbnrfifHhDYfgasaacH8akY=wiFfYdH8Gipec8Eeeu0xXdbba9frFj0=OqFfea0dXdd9vqai=hGuQ8kuc9pgc9s8qqaq=dirpe0xb9q8qiLsFr0=vr0=vr0dc8meaabaqaciGacaGaaeqabaqabeGadaaakeaacqWGdbWqdaqhaaWcbaGafm4AaSMbaebaaeaacqGGQaGkaaaaaa@303D@| and |Rki|≤|Rk−1i|
 MathType@MTEF@5@5@+=feaafiart1ev1aaatCvAUfKttLearuWrP9MDH5MBPbIqV92AaeXatLxBI9gBaebbnrfifHhDYfgasaacH8akY=wiFfYdH8Gipec8Eeeu0xXdbba9frFj0=OqFfea0dXdd9vqai=hGuQ8kuc9pgc9s8qqaq=dirpe0xb9q8qiLsFr0=vr0=vr0dc8meaabaqaciGacaGaaeqabaqabeGadaaakeaadaabdaqaaiabdkfasnaaDaaaleaacqWGRbWAaeaacqWGPbqAaaaakiaawEa7caGLiWoacqGHKjYOdaabdaqaaiabdkfasnaaDaaaleaacqWGRbWAcqGHsislcqaIXaqmaeaacqWGPbqAaaaakiaawEa7caGLiWoaaaa@3EC0@.  □

**Theorem 2. ***The first greedy algorithm gives a solution of *(*m *+ 1) lnK(K−1)2
 MathType@MTEF@5@5@+=feaafiart1ev1aaatCvAUfKttLearuWrP9MDH5MBPbIqV92AaeXatLxBI9gBaebbnrfifHhDYfgasaacH8akY=wiFfYdH8Gipec8Eeeu0xXdbba9frFj0=OqFfea0dXdd9vqai=hGuQ8kuc9pgc9s8qqaq=dirpe0xb9q8qiLsFr0=vr0=vr0dc8meaabaqaciGacaGaaeqabaqabeGadaaakeaacqqGSbaBcqqGUbGBdaWcaaqaaiabdUealjabcIcaOiabdUealjabgkHiTiabigdaXiabcMcaPaqaaiabikdaYaaaaaa@363F@*approximation*.

*Proof. *Define the *d-*th harmonic number as H(d)=∑i=1d1i
 MathType@MTEF@5@5@+=feaafiart1ev1aaatCvAUfKttLearuWrP9MDH5MBPbIqV92AaeXatLxBI9gBaebbnrfifHhDYfgasaacH8akY=wiFfYdH8Gipec8Eeeu0xXdbba9frFj0=OqFfea0dXdd9vqai=hGuQ8kuc9pgc9s8qqaq=dirpe0xb9q8qiLsFr0=vr0=vr0dc8meaabaqaciGacaGaaeqabaqabeGadaaakeaacqWGibascqGGOaakcqWGKbazcqGGPaqkcqGH9aqpdaaeWaqaamaalaaabaGaeGymaedabaGaemyAaKgaaaWcbaGaemyAaKMaeyypa0JaeGymaedabaGaemizaqganiabggHiLdaaaa@3ACF@ and *H*(0) = 0. By (2) and Lemma 3,

∑i=1m+1∑j=1K(K−1)2Cji=∑i=1m+1∑k∈Ci'(Rk−1i−Rki)1|Skc|≤∑i=1m+1∑k∈Ci'(Rk−1i−Rki)|C*|Rk−1i=∑i=1m+1∑k∈Ci'(∑l=Rki+1Rk−1i|C*|Rk−1i)≤|C*|∑i=1m+1∑k∈Ci'∑l=Rki+1Rk−1i1l     (l≤Rk−1i)=|C*|∑i=1m+1∑k∈Ci'(∑l=1Rk−1i1l−∑l=1Rki1l)≤|C*|∑i=1m+1∑k∈Ci'(H(Rk−1i)−H(Rki))≤|C*|∑i=1m+1(H(R0i)−H(R|Ci'|i))≤|C*|(m+1)max⁡{H(R0i)}     (R|Ci'|i=0 and H(0)=0)≤|C*|(m+1) ln|P|.     (H(R0i)≤H(|P|))     (3)
 MathType@MTEF@5@5@+=feaafiart1ev1aaatCvAUfKttLearuWrP9MDH5MBPbIqV92AaeXatLxBI9gBaebbnrfifHhDYfgasaacH8akY=wiFfYdH8Gipec8Eeeu0xXdbba9frFj0=OqFfea0dXdd9vqai=hGuQ8kuc9pgc9s8qqaq=dirpe0xb9q8qiLsFr0=vr0=vr0dc8meaabaqaciGacaGaaeqabaqabeGadaaakqaaeeqaamaaqahabaWaaabCaeaacqWGdbWqdaqhaaWcbaGaemOAaOgabaGaemyAaKgaaaqaaiabdQgaQjabg2da9iabigdaXaqaamaaleaameaacqWGlbWscqGGOaakcqWGlbWscqGHsislcqaIXaqmcqGGPaqkaeaacqaIYaGmaaaaniabggHiLdaaleaacqWGPbqAcqGH9aqpcqaIXaqmaeaacqWGTbqBcqGHRaWkcqaIXaqma0GaeyyeIuoakiabg2da9maaqahabaWaaabuaeaadaqadaqaaiabdkfasnaaDaaaleaacqWGRbWAcqGHsislcqaIXaqmaeaacqWGPbqAaaGccqGHsislcqWGsbGudaqhaaWcbaGaem4AaSgabaGaemyAaKgaaaGccaGLOaGaayzkaaaaleaacqWGRbWAcqGHiiIZcqWGdbWqdaqhaaadbaGaemyAaKgabaGaei4jaCcaaaWcbeqdcqGHris5aaWcbaGaemyAaKMaeyypa0JaeGymaedabaGaemyBa0Maey4kaSIaeGymaedaniabggHiLdGcdaWcbaWcbaGaeGymaedabaWaaqWaaeaacqWGtbWudaqhaaadbaGaem4AaSgabaGaem4yamgaaaWccaGLhWUaayjcSdaaaOGaeyizIm6aaabCaeaadaaeqbqaamaabmaabaGaemOuai1aa0baaSqaaiabdUgaRjabgkHiTiabigdaXaqaaiabdMgaPbaakiabgkHiTiabdkfasnaaDaaaleaacqWGRbWAaeaacqWGPbqAaaaakiaawIcacaGLPaaaaSqaaiabdUgaRjabgIGiolabdoeadnaaDaaameaacqWGPbqAaeaacqGGNaWjaaaaleqaniabggHiLdaaleaacqWGPbqAcqGH9aqpcqaIXaqmaeaacqWGTbqBcqGHRaWkcqaIXaqma0GaeyyeIuoakmaalaaabaWaaqWaaeaacqWGdbWqcqGGQaGkaiaawEa7caGLiWoaaeaacqWGsbGudaqhaaWcbaGaem4AaSMaeyOeI0IaeGymaedabaGaemyAaKgaaaaaaOqaaiabg2da9maaqahabaWaaabuaeaacqGGOaakdaaeWbqaamaalaaabaWaaqWaaeaacqWGdbWqcqGGQaGkaiaawEa7caGLiWoaaeaacqWGsbGudaqhaaWcbaGaem4AaSMaeyOeI0IaeGymaedabaGaemyAaKgaaaaaaeaacqWGSbaBcqGH9aqpcqWGsbGudaqhaaadbaGaem4AaSgabaGaemyAaKgaaSGaey4kaSIaeGymaedabaGaemOuai1aa0baaWqaaiabdUgaRjabgkHiTiabigdaXaqaaiabdMgaPbaaa0GaeyyeIuoakiabcMcaPaWcbaGaem4AaSMaeyicI4Saem4qam0aa0baaWqaaiabdMgaPbqaaiabcEcaNaaaaSqab0GaeyyeIuoaaSqaaiabdMgaPjabg2da9iabigdaXaqaaiabd2gaTjabgUcaRiabigdaXaqdcqGHris5aaGcbaGaeyizIm6aaqWaaeaacqWGdbWqcqGGQaGkaiaawEa7caGLiWoadaaeWbqaamaaqafabaWaaabCaeaadaWcaaqaaiabigdaXaqaaiabdYgaSbaaaSqaaiabdYgaSjabg2da9iabdkfasnaaDaaameaacqWGRbWAaeaacqWGPbqAaaWccqGHRaWkcqaIXaqmaeaacqWGsbGudaqhaaadbaGaem4AaSMaeyOeI0IaeGymaedabaGaemyAaKgaaaqdcqGHris5aaWcbaGaem4AaSMaeyicI4Saem4qam0aa0baaWqaaiabdMgaPbqaaiabcEcaNaaaaSqab0GaeyyeIuoaaSqaaiabdMgaPjabg2da9iabigdaXaqaaiabd2gaTjabgUcaRiabigdaXaqdcqGHris5aOGaaCzcaiaaxMaadaqadaqaaiabdYgaSjabgsMiJkabdkfasnaaDaaaleaacqWGRbWAcqGHsislcqaIXaqmaeaacqWGPbqAaaaakiaawIcacaGLPaaaaeaacqGH9aqpdaabdaqaaiabdoeadjabcQcaQaGaay5bSlaawIa7amaaqahabaWaaabuaeaacqGGOaakdaaeWbqaamaalaaabaGaeGymaedabaGaemiBaWgaaaWcbaGaemiBaWMaeyypa0JaeGymaedabaGaemOuai1aa0baaWqaaiabdUgaRjabgkHiTiabigdaXaqaaiabdMgaPbaaa0GaeyyeIuoakiabgkHiTmaaqahabaWaaSaaaeaacqaIXaqmaeaacqWGSbaBaaaaleaacqWGSbaBcqGH9aqpcqaIXaqmaeaacqWGsbGudaqhaaadbaGaem4AaSgabaGaemyAaKgaaaqdcqGHris5aaWcbaGaem4AaSMaeyicI4Saem4qam0aa0baaWqaaiabdMgaPbqaaiabcEcaNaaaaSqab0GaeyyeIuoaaSqaaiabdMgaPjabg2da9iabigdaXaqaaiabd2gaTjabgUcaRiabigdaXaqdcqGHris5aOGaeiykaKcabaGaeyizIm6aaqWaaeaacqWGdbWqcqGGQaGkaiaawEa7caGLiWoadaaeWbqaamaaqafabaGaeiikaGIaemisaGKaeiikaGIaemOuai1aa0baaSqaaiabdUgaRjabgkHiTiabigdaXaqaaiabdMgaPbaakiabcMcaPiabgkHiTiabdIeaijabcIcaOiabdkfasnaaDaaaleaacqWGRbWAaeaacqWGPbqAaaGccqGGPaqkcqGGPaqkaSqaaiabdUgaRjabgIGiolabdoeadnaaDaaameaacqWGPbqAaeaacqGGNaWjaaaaleqaniabggHiLdaaleaacqWGPbqAcqGH9aqpcqaIXaqmaeaacqWGTbqBcqGHRaWkcqaIXaqma0GaeyyeIuoaaOqaaiabgsMiJoaaemaabaGaem4qamKaeiOkaOcacaGLhWUaayjcSdWaaabCaeaacqGGOaakcqWGibascqGGOaakcqWGsbGudaqhaaWcbaGaeGimaadabaGaemyAaKgaaOGaeiykaKIaeyOeI0IaemisaGKaeiikaGIaemOuai1aa0baaSqaamaaemaabaGaem4qam0aa0baaWqaaiabdMgaPbqaaiabcEcaNaaaaSGaay5bSlaawIa7aaqaaiabdMgaPbaaaeaacqWGPbqAcqGH9aqpcqaIXaqmaeaacqWGTbqBcqGHRaWkcqaIXaqma0GaeyyeIuoakiabcMcaPiabcMcaPaqaaiabgsMiJoaaemaabaGaem4qamKaeiOkaOcacaGLhWUaayjcSdGaeiikaGIaemyBa0Maey4kaSIaeGymaeJaeiykaKIagiyBa0MaeiyyaeMaeiiEaGNaei4EaSNaemisaGKaeiikaGIaemOuai1aa0baaSqaaiabicdaWaqaaiabdMgaPbaakiabcMcaPiabc2ha9jaaxMaacaWLjaGaeiikaGIaemOuai1aa0baaSqaamaaemaabaGaem4qam0aa0baaWqaaiabdMgaPbqaaiabcEcaNaaaaSGaay5bSlaawIa7aaqaaiabdMgaPbaakiabg2da9iabicdaWiabbccaGiabbggaHjabb6gaUjabbsgaKjabbccaGiabdIeaijabcIcaOiabicdaWiabcMcaPiabg2da9iabicdaWiabcMcaPaqaaiabgsMiJoaaemaabaGaem4qamKaeiOkaOcacaGLhWUaayjcSdGaeiikaGIaemyBa0Maey4kaSIaeGymaeJaeiykaKIaeeiiaaIaeeiBaWMaeeOBa4MaeeiFaWNaemiuaaLaeiiFaWNaeiOla4IaaCzcaiaaxMaacaWLjaGaeiikaGIaemisaGKaeiikaGIaemOuai1aa0baaSqaaiabicdaWaqaaiabdMgaPbaakiabcMcaPiabgsMiJkabdIeaijabcIcaOiabcYha8jabdcfaqjabcYha8jabcMcaPiabcMcaPiaaxMaacaWLjaWaaeWaaeaacqaIZaWmaiaawIcacaGLPaaaaaaa@DEEA@

By (1) and (3), we get

|C'||C*|≤(m+1) ln |P| = (m+1) lnK(K−1)2.
 MathType@MTEF@5@5@+=feaafiart1ev1aaatCvAUfKttLearuWrP9MDH5MBPbIqV92AaeXatLxBI9gBaebbnrfifHhDYfgasaacH8akY=wiFfYdH8Gipec8Eeeu0xXdbba9frFj0=OqFfea0dXdd9vqai=hGuQ8kuc9pgc9s8qqaq=dirpe0xb9q8qiLsFr0=vr0=vr0dc8meaabaqaciGacaGaaeqabaqabeGadaaakeaadaWcaaqaaiabcYha8jabdoeadjabcEcaNiabcYha8bqaaiabcYha8jabdoeadjabcQcaQiabcYha8baacqGHKjYOcqGGOaakcqWGTbqBcqGHRaWkcqaIXaqmcqGGPaqkcqqGGaaicqqGSbaBcqqGUbGBcqqGGaaicqqG8baFcqWGqbaucqGG8baFcqqGGaaicqqG9aqpcqqGGaaicqqGOaakcqWGTbqBcqGHRaWkcqaIXaqmcqGGPaqkcqqGGaaicqqGSbaBcqqGUbGBdaWcaaqaaiabdUealjabcIcaOiabdUealjabgkHiTiabigdaXiabcMcaPaqaaiabikdaYaaacqGGUaGlaaa@5853@

### The second greedy algorithm

This section describes the second greedy algorithm which returns a solution of better approximation than that of the first greedy algorithm. Let *R*_*i *_be the set of uncovered grids at the *i*-th row. Unlike the row-by-row manner of the first greedy algorithm, this algorithm greedily selects a SNP that covers most uncovered grids in the table (i.e., its reformulated set *S' *maximizing |*S' *∩ (*R*_1 _∪ ... ∪ *R*_*m*+1_)|). Let *T *be the collection of *R*_*i *_(i.e., *T *is the set of all uncovered grids in the table). If the grids in the *i*-th row are all covered (i.e., *R*_*i *_= *φ*), *R*_*i *_is removed from *T*. This algorithm runs until *T *= *φ *(i.e., all grids in the table are covered).

**Figure 9:**
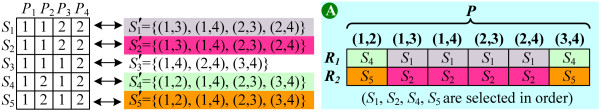
**An example of the second greedy algorithm**. The SNPs *S*_1_, *S*_2_, *S*_4_, and *S*_5 _are selected by the second greedy algorithm. (A) The table that stores each selected SNP.

Figure 9 illustrates an example for this algorithm with *m *set to 1. The SNPs *S*_1_, *S*_2_, *S*_4_, and *S*_5 _are selected in order. Since this algorithm runs until all grids are covered, the set of SNPs in this table is able to tolerate *m *missing tag SNPs. The pseudo code of this algorithm is given below.

**Algorithm: **SECOND-GREEDY-ALGORITHM (*C*, *P*, *m*)

1 *R*_*i *_← *P*, ∀ *i *∈ [1, *m *+ 1]

2 *T *← {R_1_, R_2_,... ,*R*_*m*+1_}

3 *C' *← *φ*

4 **while ***T *≠ *φ ***do**

5    select and remove a SNP *S *from *C *that maximizes |*S' *∩ (*R*_1 _∪ ... ∪ *R*_*m*+1_)|

6    *C' *← *C' *∪ *S*

7    **for **each *R*_*i *_∈ *T ***and ***S' *≠ *ø ***do**

8       S_*tmp *_← *S' *∩ *R*_*i *_// *S*_*tmp *_is a temporary variable for holding the result of *S' *∩ *R*_*i*_

9       *R*_*i *_← *R*_*i *_- *S*_*tmp*_

10       *S' *← *S' *- *S*_*tmp*_

11       **if ***R*_*i *_= *φ ***then ***T *← *T *- *R*_*i*_

12    **endfor**

13 **end while**

14 **return ***C'*

The time complexity of this algorithm is analyzed as follows. At Line 4, the number of iterations of the loop is bounded by *O*(|*T*|) = *O*(*m*|*P*|). Within the loop, Line 5 takes *O*(|*C*||*P*|) time because we need to check each SNP in *C *and examine if it can cover any uncovered grid in each column. The inner loop (Lines 7–12) is bounded by *O*(|*S'*|) <*O*(|*P*|). Thus, the running time of this program is *O*(*m*|*C*||*P*|^2^).

We now evaluate the solution returned by the second greedy algorithm. Let *C' *and C* be the set of SNPs selected by this algorithm and the optimal solution, respectively. Let |Skc
 MathType@MTEF@5@5@+=feaafiart1ev1aaatCvAUfKttLearuWrP9MDH5MBPbIqV92AaeXatLxBI9gBaebbnrfifHhDYfgasaacH8akY=wiFfYdH8Gipec8Eeeu0xXdbba9frFj0=OqFfea0dXdd9vqai=hGuQ8kuc9pgc9s8qqaq=dirpe0xb9q8qiLsFr0=vr0=vr0dc8meaabaqaciGacaGaaeqabaqabeGadaaakeaacqWGtbWudaqhaaWcbaGaem4AaSgabaGaem4yamgaaaaa@30B8@| be the number of grids in the table covered by the *k*-th selected SNP. For example (see Figure 9), |S2c
 MathType@MTEF@5@5@+=feaafiart1ev1aaatCvAUfKttLearuWrP9MDH5MBPbIqV92AaeXatLxBI9gBaebbnrfifHhDYfgasaacH8akY=wiFfYdH8Gipec8Eeeu0xXdbba9frFj0=OqFfea0dXdd9vqai=hGuQ8kuc9pgc9s8qqaq=dirpe0xb9q8qiLsFr0=vr0=vr0dc8meaabaqaciGacaGaaeqabaqabeGadaaakeaacqWGtbWudaqhaaWcbaGaeGOmaidabaGaem4yamgaaaaa@304B@| = 4 since the second selected SNP (i.e., *S*_2_) covers four grids in the table. Define *T*_*k *_as the number of uncovered grids in the table before the *k*-th iteration. We have the following lemma similar to Lemma 3.

**Lemma 4. ***The k-th selected SNP has *|Skc|≥Tk−1|C*|
 MathType@MTEF@5@5@+=feaafiart1ev1aaatCvAUfKttLearuWrP9MDH5MBPbIqV92AaeXatLxBI9gBaebbnrfifHhDYfgasaacH8akY=wiFfYdH8Gipec8Eeeu0xXdbba9frFj0=OqFfea0dXdd9vqai=hGuQ8kuc9pgc9s8qqaq=dirpe0xb9q8qiLsFr0=vr0=vr0dc8meaabaqaciGacaGaaeqabaqabeGadaaakeaadaabdaqaaiabdofatnaaDaaaleaacqWGRbWAaeaacqWGJbWyaaaakiaawEa7caGLiWoacqGHLjYSdaWcaaqaaiabdsfaunaaBaaaleaacqWGRbWAcqGHsislcqaIXaqmaeqaaaGcbaGaeiiFaWNaem4qamKaeiOkaOIaeiiFaWhaaaaa@3F48@.

*Proof. *The proof is similar to that of Lemma 3. Let Ck¯*
 MathType@MTEF@5@5@+=feaafiart1ev1aaatCvAUfKttLearuWrP9MDH5MBPbIqV92AaeXatLxBI9gBaebbnrfifHhDYfgasaacH8akY=wiFfYdH8Gipec8Eeeu0xXdbba9frFj0=OqFfea0dXdd9vqai=hGuQ8kuc9pgc9s8qqaq=dirpe0xb9q8qiLsFr0=vr0=vr0dc8meaabaqaciGacaGaaeqabaqabeGadaaakeaacqWGdbWqdaqhaaWcbaGafm4AaSMbaebaaeaacqGGQaGkaaaaaa@303D@ be the set of remaining SNPs in C* which has not been selected before the *k*-th iteration. We claim that there exists a SNP in Ck¯*
 MathType@MTEF@5@5@+=feaafiart1ev1aaatCvAUfKttLearuWrP9MDH5MBPbIqV92AaeXatLxBI9gBaebbnrfifHhDYfgasaacH8akY=wiFfYdH8Gipec8Eeeu0xXdbba9frFj0=OqFfea0dXdd9vqai=hGuQ8kuc9pgc9s8qqaq=dirpe0xb9q8qiLsFr0=vr0=vr0dc8meaabaqaciGacaGaaeqabaqabeGadaaakeaacqWGdbWqdaqhaaWcbaGafm4AaSMbaebaaeaacqGGQaGkaaaaaa@303D@ which can cover at least Tk|Ck¯*|
 MathType@MTEF@5@5@+=feaafiart1ev1aaatCvAUfKttLearuWrP9MDH5MBPbIqV92AaeXatLxBI9gBaebbnrfifHhDYfgasaacH8akY=wiFfYdH8Gipec8Eeeu0xXdbba9frFj0=OqFfea0dXdd9vqai=hGuQ8kuc9pgc9s8qqaq=dirpe0xb9q8qiLsFr0=vr0=vr0dc8meaabaqaciGacaGaaeqabaqabeGadaaakeaadaWcaaqaaiabdsfaunaaBaaaleaacqWGRbWAaeqaaaGcbaWaaqWaaeaacqWGdbWqdaqhaaWcbaGafm4AaSMbaebaaeaacqGGQaGkaaaakiaawEa7caGLiWoaaaaaaa@363F@ grids in the table. Otherwise, we can get the same contradiction (i.e., C* fails to cover all grids) as in Lemma 3. Since |C*| ≥ |Ck¯*
 MathType@MTEF@5@5@+=feaafiart1ev1aaatCvAUfKttLearuWrP9MDH5MBPbIqV92AaeXatLxBI9gBaebbnrfifHhDYfgasaacH8akY=wiFfYdH8Gipec8Eeeu0xXdbba9frFj0=OqFfea0dXdd9vqai=hGuQ8kuc9pgc9s8qqaq=dirpe0xb9q8qiLsFr0=vr0=vr0dc8meaabaqaciGacaGaaeqabaqabeGadaaakeaacqWGdbWqdaqhaaWcbaGafm4AaSMbaebaaeaacqGGQaGkaaaaaa@303D@| and *T*_*k*-1 _≤ *T*_*k*_, we have |Skc|≥Tk−1|C*|
 MathType@MTEF@5@5@+=feaafiart1ev1aaatCvAUfKttLearuWrP9MDH5MBPbIqV92AaeXatLxBI9gBaebbnrfifHhDYfgasaacH8akY=wiFfYdH8Gipec8Eeeu0xXdbba9frFj0=OqFfea0dXdd9vqai=hGuQ8kuc9pgc9s8qqaq=dirpe0xb9q8qiLsFr0=vr0=vr0dc8meaabaqaciGacaGaaeqabaqabeGadaaakeaadaabdaqaaiabdofatnaaDaaaleaacqWGRbWAaeaacqWGJbWyaaaakiaawEa7caGLiWoacqGHLjYSdaWcaaqaaiabdsfaunaaBaaaleaacqWGRbWAcqGHsislcqaIXaqmaeqaaaGcbaGaeiiFaWNaem4qamKaeiOkaOIaeiiFaWhaaaaa@3F48@.     □

**Theorem 3. ***The second greedy algorithm gives a solution of *ln((m+1)K(K−1)2)
 MathType@MTEF@5@5@+=feaafiart1ev1aaatCvAUfKttLearuWrP9MDH5MBPbIqV92AaeXatLxBI9gBaebbnrfifHhDYfgasaacH8akY=wiFfYdH8Gipec8Eeeu0xXdbba9frFj0=OqFfea0dXdd9vqai=hGuQ8kuc9pgc9s8qqaq=dirpe0xb9q8qiLsFr0=vr0=vr0dc8meaabaqaciGacaGaaeqabaqabeGadaaakeaacqqGSbaBcqqGUbGBcqqGOaakcqqGOaakcqWGTbqBcqGHRaWkcqaIXaqmcqGGPaqkdaWcaaqaaiabdUealjabcIcaOiabdUealjabgkHiTiabigdaXiabcMcaPaqaaiabikdaYaaacqGGPaqkaaa@3CD6@*approximation*.

**Figure 10:**
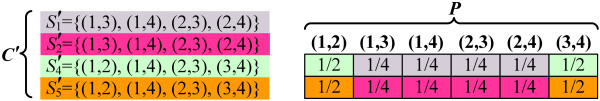
**Analysis of the second greedy algorithm**. This figure shows the cost Cji
 MathType@MTEF@5@5@+=feaafiart1ev1aaatCvAUfKttLearuWrP9MDH5MBPbIqV92AaeXatLxBI9gBaebbnrfifHhDYfgasaacH8akY=wiFfYdH8Gipec8Eeeu0xXdbba9frFj0=OqFfea0dXdd9vqai=hGuQ8kuc9pgc9s8qqaq=dirpe0xb9q8qiLsFr0=vr0=vr0dc8meaabaqaciGacaGaaeqabaqabeGadaaakeaacqWGdbWqdaqhaaWcbaGaemOAaOgabaGaemyAaKgaaaaa@30A2@ of each grid for the second greedy algorithm.

*Proof. *Each grid at the *i*-th row and *j*-th column is assigned a cost Cji=1|Skc|
 MathType@MTEF@5@5@+=feaafiart1ev1aaatCvAUfKttLearuWrP9MDH5MBPbIqV92AaeXatLxBI9gBaebbnrfifHhDYfgasaacH8akY=wiFfYdH8Gipec8Eeeu0xXdbba9frFj0=OqFfea0dXdd9vqai=hGuQ8kuc9pgc9s8qqaq=dirpe0xb9q8qiLsFr0=vr0=vr0dc8meaabaqaciGacaGaaeqabaqabeGadaaakeaacqWGdbWqdaqhaaWcbaGaemOAaOgabaGaemyAaKgaaOGaeyypa0ZaaSaaaeaacqaIXaqmaeaadaabdaqaaiabdofatnaaDaaaleaacqWGRbWAaeaacqWGJbWyaaaakiaawEa7caGLiWoaaaaaaa@39E8@ (see Figure 10) if it is covered by the *k*-th selected SNP. The sum of Cji
 MathType@MTEF@5@5@+=feaafiart1ev1aaatCvAUfKttLearuWrP9MDH5MBPbIqV92AaeXatLxBI9gBaebbnrfifHhDYfgasaacH8akY=wiFfYdH8Gipec8Eeeu0xXdbba9frFj0=OqFfea0dXdd9vqai=hGuQ8kuc9pgc9s8qqaq=dirpe0xb9q8qiLsFr0=vr0=vr0dc8meaabaqaciGacaGaaeqabaqabeGadaaakeaacqWGdbWqdaqhaaWcbaGaemOAaOgabaGaemyAaKgaaaaa@30A2@ for each grid is

|C'| = ∑i=1m+1∑j=1K(K−1)2Cji=∑k=1|C'|(Tk−1−Tk)1|Skc|(see (1) and (2))≤∑k=1|C'|(Tk−1−Tk)|C*|TK−1(by Lemma 4)≤|C*|(H(T0)−H(T|C'|))(see the proof in Theorem 2)≤|C*|ln((m+1)|P|).     (4)
 MathType@MTEF@5@5@+=feaafiart1ev1aaatCvAUfKttLearuWrP9MDH5MBPbIqV92AaeXatLxBI9gBaebbnrfifHhDYfgasaacH8akY=wiFfYdH8Gipec8Eeeu0xXdbba9frFj0=OqFfea0dXdd9vqai=hGuQ8kuc9pgc9s8qqaq=dirpe0xb9q8qiLsFr0=vr0=vr0dc8meaabaqaciGacaGaaeqabaqabeGadaaakeaafaqaaeabeaaaaaqaaiabcYha8jabdoeadjabcEcaNiabcYha8jabbccaGiabb2da9iabbccaGmaaqahabaWaaabCaeaacqWGdbWqdaqhaaWcbaGaemOAaOgabaGaemyAaKgaaaqaaiabdQgaQjabg2da9iabigdaXaqaamaaleaameaacqWGlbWscqGGOaakcqWGlbWscqGHsislcqaIXaqmcqGGPaqkaeaacqaIYaGmaaaaniabggHiLdaaleaacqWGPbqAcqGH9aqpcqaIXaqmaeaacqWGTbqBcqGHRaWkcqaIXaqma0GaeyyeIuoaaOqaaiabg2da9aqaamaaqahabaGaeiikaGIaemivaq1aaSbaaSqaaiabdUgaRjabgkHiTiabigdaXaqabaGccqGHsislcqWGubavdaWgaaWcbaGaem4AaSgabeaakiabcMcaPmaalaaabaGaeGymaedabaWaaqWaaeaacqWGtbWudaqhaaWcbaGaem4AaSgabaGaem4yamgaaaGccaGLhWUaayjcSdaaaaWcbaGaem4AaSMaeyypa0JaeGymaedabaGaeiiFaWNaem4qamKaei4jaCIaeiiFaWhaniabggHiLdaakeaacqGGOaakcqqGZbWCcqqGLbqzcqqGLbqzcqqGGaaicqqGOaakcqqGXaqmcqqGPaqkcqqGGaaicqqGHbqycqqGUbGBcqqGKbazcqqGGaaicqqGOaakcqqGYaGmcqqGPaqkcqqGPaqkaeaaaeaacqGHKjYOaeaadaaeWbqaamXvP5wqSXMqHnxAJn0BKvguHDwzZbqegyvzYrwyUfgaiqaacaWFOaGaemivaq1aaSbaaSqaaiabdUgaRjabgkHiTiabigdaXaqabaGccqGHsislcqWGubavdaWgaaWcbaGaem4AaSgabeaakiabcMcaPaWcbaGaem4AaSMaeyypa0JaeGymaedabaGaeeiFaWhceiGaa43qaiaa=DcacqqG8baFa0GaeyyeIuoakmaalaaabaGaeiiFaWNaem4qamKaeiOkaOIaeiiFaWhabaGaemivaq1aaSbaaSqaaiabdUealjabgkHiTiabigdaXaqabaaaaaGcbaGaeiikaGIaeeOyaiMaeeyEaKNaeeiiaaIaeeitaWKaeeyzauMaeeyBa0MaeeyBa0MaeeyyaeMaeeiiaaIaeeinaqJaeeykaKcabaaabaGaeyizImkabaGaeiiFaWNaem4qamKaeiOkaOIaeiiFaWNaeiikaGIaemisaGKaeiikaGIaemivaq1aaSbaaSqaaiabicdaWaqabaGccqGGPaqkcqGHsislcqWGibascqGGOaakcqWGubavdaWgaaWcbaGaeiiFaWNaem4qamKaei4jaCIaeiiFaWhabeaakiabcMcaPiabcMcaPaqaaiabbIcaOiabbohaZjabbwgaLjabbwgaLjabbccaGiabbsha0jabbIgaOjabbwgaLjabbccaGiabbchaWjabbkhaYjabb+gaVjabb+gaVjabbAgaMjabbccaGiabbMgaPjabb6gaUjabbccaGiabbsfaujabbIgaOjabbwgaLjabb+gaVjabbkhaYjabbwgaLjabb2gaTjabbccaGiabbkdaYiabbMcaPaqaaaqaaiabgsMiJcqaaiabbYha8jabdoeadjabcQcaQiabcYha8jabbYgaSjabb6gaUjabcIcaOiabcIcaOiabd2gaTjabgUcaRiabigdaXiabcMcaPiabcYha8jabdcfaqjabcYha8jabcMcaPiabc6caUaqaaiaaxMaacaWLjaGaaCzcaiaaxMaacaWLjaWaaeWaaeaacqqG0aanaiaawIcacaGLPaaaaaaaaa@073E@

By (4), we have

|C'||C*|≤ln⁡((m+1)|P|)=ln⁡((m+1)K(K−1)2).
 MathType@MTEF@5@5@+=feaafiart1ev1aaatCvAUfKttLearuWrP9MDH5MBPbIqV92AaeXatLxBI9gBaebbnrfifHhDYfgasaacH8akY=wiFfYdH8Gipec8Eeeu0xXdbba9frFj0=OqFfea0dXdd9vqai=hGuQ8kuc9pgc9s8qqaq=dirpe0xb9q8qiLsFr0=vr0=vr0dc8meaabaqaciGacaGaaeqabaqabeGadaaakeaadaWcaaqaaiabcYha8jabdoeadjabcEcaNiabcYha8bqaaiabcYha8jabdoeadjabcQcaQiabcYha8baacqGHKjYOcyGGSbaBcqGGUbGBcqGGOaakcqGGOaakcqWGTbqBcqGHRaWkcqaIXaqmcqGGPaqkcqGG8baFcqWGqbaucqGG8baFcqGGPaqkcqGH9aqpcyGGSbaBcqGGUbGBcqGGOaakcqGGOaakcqWGTbqBcqGHRaWkcqaIXaqmcqGGPaqkdaWcaaqaaiabdUealjabcIcaOiabdUealjabgkHiTiabigdaXiabcMcaPaqaaiabikdaYaaacqGGPaqkcqGGUaGlaaa@57E3@

### The iterative LP-relaxation algorithm

In practice, a probabilistic approach is sometimes more useful since the randomization can explore different solutions. In this section, we reformulate the MRTS problem to an *Integer Programming *(IP) problem. Based on the IP problem, we propose an iterative Linear Programming (LP)-relaxation algorithm. The iterative LP-relaxation algorithm is described below.

**Step 1. **Given a haplotype block containing *N *SNPs and *K *haplotype patterns. Let {*x*_1_,*x*_2_, ...,*x*_*N*_} be the set of integer variables for the *N *SNPs, where *x*_*k *_= 1 if the SNP *S*_*k *_is selected and *x*_*k *_= 0 otherwise. Define *D*(*P*_*i*_, *P*_*j*_) as the set of SNPs which are able to distinguish *P*_*i *_and *P*_*j *_patterns. By Lemma 1, to allow at most *m *missing SNPs, each pair of patterns must be distinguished by at least (*m *+ 1) SNPs. Therefore, for each set *D*(*P*_*i*_, *P*_*j*_), at least (*m *+ 1) SNPs have to be selected to distinguish *P*_*i *_and *P*_*j *_patterns. As a consequence, the MRTS problem can be formulated as the following IP problem:

Minimize∑k=1NxkSubjectto ∑k∈D(Pi,Pj)xk=0 or 1.xk≥m+1,for all 1≤i<j≤K,     (5)
 MathType@MTEF@5@5@+=feaafiart1ev1aaatCvAUfKttLearuWrP9MDH5MBPbIqV92AaeXatLxBI9gBaebbnrfifHhDYfgasaacH8akY=wiFfYdH8Gipec8Eeeu0xXdbba9frFj0=OqFfea0dXdd9vqai=hGuQ8kuc9pgc9s8qqaq=dirpe0xb9q8qiLsFr0=vr0=vr0dc8meaabaqaciGacaGaaeqabaqabeGadaaakeaafaqaaeGacaaabaacbeGae8xta0Kae8xAaKMae8NBa4Mae8xAaKMae8xBa0Mae8xAaKMae8NEaONae8xzaugabaWaaabCaeaacqWG4baEdaWgaaWcbaGaem4AaSgabeaaaeaacqWGRbWAcqGH9aqpcqaIXaqmaeaacqWGobGta0GaeyyeIuoaaOqaaiab=nfatjab=vha1jab=jgaIjab=PgaQjab=vgaLjab=ngaJjab=rha0jab=bcaGiab=rha0jab=9gaVbqaaiabbccaGmaaqafabaGaemiEaG3aaSbaaSqaaiabdUgaRbqabaGccqGHLjYScqWGTbqBcqGHRaWkcqaIXaqmcqGGSaalcaWLjaGaeeOzayMaee4Ba8MaeeOCaiNaeeiiaaIaeeyyaeMaeeiBaWMaeeiBaWMaeeiiaaIaeeymaeJaeyizImQaemyAaKMaeyipaWJaemOAaOMaeyizImQaem4saSKaeiilaWcaleaafaqabeGabaaabaGaem4AaSMaeyicI4SaemiraqKaeiikaGIaemiuaa1aaSbaaWqaaiabdMgaPbqabaWccqGGSaalcqWGqbaudaWgaaadbaGaemOAaOgabeaaliabcMcaPaqaaiabdIha4naaBaaameaacqWGRbWAaeqaaSGaeyypa0JaeGimaaJaeeiiaaIaee4Ba8MaeeOCaiNaeeiiaaIaeeymaeJaeeOla4caaaqab0GaeyyeIuoakiaaxMaacaWLjaWaaeWaaeaacqaI1aqnaiaawIcacaGLPaaaaaaaaa@894F@

**Step 2. **Since solving the IP problem is NP-hard [6], we relax the integer constraint of *x*_*k*_, and the IP problem becomes an LP problem defined as follows:

Minimize∑k=1NykSubjectto∑k∈D(Pi,Pj)0  ≤  yk  ≤  1.yk≥m+1,     (6)
 MathType@MTEF@5@5@+=feaafiart1ev1aaatCvAUfKttLearuWrP9MDH5MBPbIqV92AaeXatLxBI9gBaebbnrfifHhDYfgasaacH8akY=wiFfYdH8Gipec8Eeeu0xXdbba9frFj0=OqFfea0dXdd9vqai=hGuQ8kuc9pgc9s8qqaq=dirpe0xb9q8qiLsFr0=vr0=vr0dc8meaabaqaciGacaGaaeqabaqabeGadaaakeaafaqaaeGacaaabaWexLMBbXgBcf2CPn2qVrwzqf2zLnharyGvLjhzH5wyaGabbiaa=1eacaWFPbGaa8NBaiaa=LgacaWFTbGaa8xAaiaa=PhacaWFLbaabaWaaabCaeaacqWG5bqEdaWgaaWcbaGaem4AaSgabeaaaeaacqWGRbWAcqGH9aqpcqaIXaqmaeaacqWGobGta0GaeyyeIuoaaOqaaiaa=nfacaWF1bGaa8Nyaiaa=PgacaWFLbGaa83yaiaa=rhacaWFGaGaa8hDaiaa=9gaaeaadaaeqbqaaiabdMha5naaBaaaleaacqWGRbWAaeqaaOGaeyyzImRaemyBa0Maey4kaSIaeGymaedaleaafaqabeGabaaabaGaem4AaSMaeyicI4SaemiraqKaeiikaGIaemiuaa1aaSbaaWqaaiabdMgaPbqabaWccqGGSaalcqWGqbaudaWgaaadbaGaemOAaOgabeaaliabcMcaPaqaaiabicdaWiaaykW7caaMc8UaeyizImQaaGPaVlaaykW7cqWG5bqEdaWgaaadbaGaem4AaSgabeaaliaaykW7caaMc8UaeyizImQaaGPaVlaaykW7cqaIXaqmcqGGUaGlaaaabeqdcqGHris5aOGaeiilaWIaaCzcaiabbAgaMjabb+gaVjabbkhaYjabbccaGiabbggaHjabbYgaSjabbYgaSjabbccaGiabbgdaXiabgsMiJkabdMgaPjabgYda8iabdQgaQjabgsMiJkabdUealjabcYcaSiaaxMaacaWLjaWaaeWaaeaacqaI2aGnaiaawIcacaGLPaaaaaaaaa@961B@

The above LP problem can be solved in polynomial time by efficient algorithms such as the interior point method (Forsgren *et al*., 2002) [5].

**Step 3. **Let {*y*_1_, *y*_2_, ..., *y*_*N*_} be the set of linear solutions obtained from (6), where 0 ≤ *y*_*k *_≤ 1. We construct the corresponding integer solutions {*x*_1_, *x*_2_, ..., *x*_*N*_} by the following randomized rounding method:

Assign{xk=1 with probability yk,xk=0 with probability 1-yk.
 MathType@MTEF@5@5@+=feaafiart1ev1aaatCvAUfKttLearuWrP9MDH5MBPbIqV92AaeXatLxBI9gBaebbnrfifHhDYfgasaacH8akY=wiFfYdH8Gipec8Eeeu0xXdbba9frFj0=OqFfea0dXdd9vqai=hGuQ8kuc9pgc9s8qqaq=dirpe0xb9q8qiLsFr0=vr0=vr0dc8meaabaqaciGacaGaaeqabaqabeGadaaakeaacqqGbbqqcqqGZbWCcqqGZbWCcqqGPbqAcqqGNbWzcqqGUbGBdaGabaqaauaabaqaceaaaeaacqWG4baEdaWgaaWcbaGaem4AaSgabeaakiabg2da9iabigdaXiabbccaGiabbEha3jabbMgaPjabbsha0jabbIgaOjabbccaGiabbchaWjabbkhaYjabb+gaVjabbkgaIjabbggaHjabbkgaIjabbMgaPjabbYgaSjabbMgaPjabbsha0jabbMha5jabbccaGiabdMha5naaBaaaleaacqWGRbWAaeqaaOGaeiilaWcabaGaemiEaG3aaSbaaSqaaiabdUgaRbqabaGccqGH9aqpcqaIWaamcqqGGaaicqqG3bWDcqqGPbqAcqqG0baDcqqGObaAcqqGGaaicqqGWbaCcqqGYbGCcqqGVbWBcqqGIbGycqqGHbqycqqGIbGycqqGPbqAcqqGSbaBcqqGPbqAcqqG0baDcqqG5bqEcqqGGaaicqqGXaqmcqqGTaqlcqWG5bqEdaWgaaWcbaGaem4AaSgabeaakiabc6caUaaaaiaawUhaaaaa@776C@

Note that the constructed integer solutions do not necessary satisfy all inequalities in (5). The randomized rounding method simply assigns *x*_*k *_to 1 or 0 using the value of *y*_*k *_as the likelihood, regardless of the inequalities in (5).

**Step 4. **We check whether the integer solutions constructed in Step 3 satisfy all inequalities in (5) or not.

**Case 1. **If some inequalities in (5) are still unsatisfied, we repeat Steps 1, 2, and 3 only for those unsatisfied inequalities until all of them are satisfied.

**Case 2. **If all inequalities in (5) are satisfied, we construct a final solution by setting *x*_*k *_= 1 if *x*_*k *_is assigned to 1 in any one of the iterations and setting *x*_*k *_= 0 otherwise.

We now evaluate the solution returned by the iterative LP-relaxation algorithm. The selection of each SNP is considered as a *Bernoulli *random variable *x*_*k *_taking values 1 (or 0) with probability *y*_*k *_(or 1 - *y*_*k*_). Let *X*_*i*,*j *_be the sum of random variables in one inequality of (5), i.e.,

Xi,j=∑k∈D{Pi,Pj}xk.
 MathType@MTEF@5@5@+=feaafiart1ev1aaatCvAUfKttLearuWrP9MDH5MBPbIqV92AaeXatLxBI9gBaebbnrfifHhDYfgasaacH8akY=wiFfYdH8Gipec8Eeeu0xXdbba9frFj0=OqFfea0dXdd9vqai=hGuQ8kuc9pgc9s8qqaq=dirpe0xb9q8qiLsFr0=vr0=vr0dc8meaabaqaciGacaGaaeqabaqabeGadaaakeaacqWGybawdaWgaaWcbaGaemyAaKMaeiilaWIaemOAaOgabeaakiabg2da9maaqafabaGaemiEaG3aaSbaaSqaaiabdUgaRbqabaGccqGGUaGlaSqaaiabdUgaRjabgIGiolabdseaenaacmaabaGaemiuaa1aaSbaaWqaaiabdMgaPbqabaWccqGGSaalcqWGqbaudaWgaaadbaGaemOAaOgabeaaaSGaay5Eaiaaw2haaaqab0GaeyyeIuoaaaa@454F@

By (6), the expected value of *X*_*i*,*j *_(after randomized rounding) is

E[Xi,j]=∑k∈D{Pi,Pj}E[xk]=∑k∈D{Pi,Pj}yk≥m+1.     (7)
 MathType@MTEF@5@5@+=feaafiart1ev1aaatCvAUfKttLearuWrP9MDH5MBPbIqV92AaeXatLxBI9gBaebbnrfifHhDYfgasaacH8akY=wiFfYdH8Gipec8Eeeu0xXdbba9frFj0=OqFfea0dXdd9vqai=hGuQ8kuc9pgc9s8qqaq=dirpe0xb9q8qiLsFr0=vr0=vr0dc8meaabaqaciGacaGaaeqabaqabeGadaaakeaafaqaaeGadaaabaGaemyrauKaei4waSLaemiwaG1aaSbaaSqaaiabdMgaPjabcYcaSiabdQgaQbqabaGccqGGDbqxaeaacqGH9aqpaeaadaaeqbqaaiabdweafjabcUfaBjabdIha4naaBaaaleaacqWGRbWAaeqaaOGaeiyxa0Laeyypa0ZaaabuaeaacqWG5bqEdaWgaaWcbaGaem4AaSgabeaaaeaacqWGRbWAcqGHiiIZcqWGebarcqGG7bWEcqWGqbaudaWgaaadbaGaemyAaKgabeaaliabcYcaSiabdcfaqnaaBaaameaacqWGQbGAaeqaaSGaeiyFa0habeqdcqGHris5aaWcbaGaem4AaSMaeyicI4SaemiraqKaei4EaSNaemiuaa1aaSbaaWqaaiabdMgaPbqabaWccqGGSaalcqWGqbaudaWgaaadbaGaemOAaOgabeaaliabc2ha9bqab0GaeyyeIuoaaOqaaaqaaiabgwMiZcqaaiabd2gaTjabgUcaRiabigdaXiabc6caUiaaxMaacaWLjaGaaCzcaiaaxMaacaWLjaWaaeWaaeaacqaI3aWnaiaawIcacaGLPaaaaaaaaa@6B7C@

**Lemma 5. ***The probability that an inequality in *(5) *is not satisfied after randomized rounding is less than *e−12(m+1)
 MathType@MTEF@5@5@+=feaafiart1ev1aaatCvAUfKttLearuWrP9MDH5MBPbIqV92AaeXatLxBI9gBaebbnrfifHhDYfgasaacH8akY=wiFfYdH8Gipec8Eeeu0xXdbba9frFj0=OqFfea0dXdd9vqai=hGuQ8kuc9pgc9s8qqaq=dirpe0xb9q8qiLsFr0=vr0=vr0dc8meaabaqaciGacaGaaeqabaqabeGadaaakeaacqWGLbqzdaahaaWcbeqaaiabgkHiTmaaleaameaacqaIXaqmaeaacqaIYaGmcqGGOaakcqWGTbqBcqGHRaWkcqaIXaqmcqGGPaqkaaaaaaaa@3601@.

*Proof. *The probability that an inequality in (5) is not satisfied is *P*[*X*_*i*,*j *_<*m *+ 1] = *P*[*X*_*i*,*j *_≤ *m*]. By the *Chernoff *bound (i.e., *P*[*X *≤ (1 - *θ*) *E*[*X*]] ≤ e−θ2E[X]2
 MathType@MTEF@5@5@+=feaafiart1ev1aaatCvAUfKttLearuWrP9MDH5MBPbIqV92AaeXatLxBI9gBaebbnrfifHhDYfgasaacH8akY=wiFfYdH8Gipec8Eeeu0xXdbba9frFj0=OqFfea0dXdd9vqai=hGuQ8kuc9pgc9s8qqaq=dirpe0xb9q8qiLsFr0=vr0=vr0dc8meaabaqaciGacaGaaeqabaqabeGadaaakeaacqWGLbqzdaahaaWcbeqaaiabgkHiTmaaleaameaacqaH4oqCdaahaaqabeaacqaIYaGmaaGaemyrauKaei4waSLaemiwaGLaeiyxa0fabaGaeGOmaidaaaaaaaa@37C0@), we have

P[Xi,j≤m]≤e−(E[Xi,j]−m)22E[Xi,j].     (8)
 MathType@MTEF@5@5@+=feaafiart1ev1aaatCvAUfKttLearuWrP9MDH5MBPbIqV92AaeXatLxBI9gBaebbnrfifHhDYfgasaacH8akY=wiFfYdH8Gipec8Eeeu0xXdbba9frFj0=OqFfea0dXdd9vqai=hGuQ8kuc9pgc9s8qqaq=dirpe0xb9q8qiLsFr0=vr0=vr0dc8meaabaqaciGacaGaaeqabaqabeGadaaakeaacqWGqbaucqGGBbWwcqWGybawdaWgaaWcbaGaemyAaKMaeiilaWIaemOAaOgabeaakiabgsMiJkabd2gaTjabc2faDjabgsMiJkabdwgaLnaaCaaaleqabaGaeyOeI0YaaSqaaWqaaiabcIcaOiabdweafjabcUfaBjabdIfaynaaBaaabaGaemyAaKMaeiilaWIaemOAaOgabeaacqGGDbqxcqGHsislcqWGTbqBcqGGPaqkdaahaaqabeaacqaIYaGmaaaabaGaeGOmaiJaemyrauKaei4waSLaemiwaG1aaSbaaeaacqWGPbqAcqGGSaalcqWGQbGAaeqaaiabc2faDbaaaaGccqGGUaGlcaWLjaGaaCzcamaabmaabaGaeGioaGdacaGLOaGaayzkaaaaaa@5880@

By (7), we know *E*[*X*_*i*,*j*_] ≤ *m *+ 1. Since the right-hand side of (8) decreases when *E*[*X*_*i*,*j*_] > *m*, we can replace *E*[*X*_*i*_,_*j*_] with (*m *+ 1) to obtain an upper bound, i.e.,

P[Xi,j≤m]≤e−(E[Xi,j]−m)22E[Xi,j]≤e−12(m+1).≤e−(m+1−m)22(m+1)
 MathType@MTEF@5@5@+=feaafiart1ev1aaatCvAUfKttLearuWrP9MDH5MBPbIqV92AaeXatLxBI9gBaebbnrfifHhDYfgasaacH8akY=wiFfYdH8Gipec8Eeeu0xXdbba9frFj0=OqFfea0dXdd9vqai=hGuQ8kuc9pgc9s8qqaq=dirpe0xb9q8qiLsFr0=vr0=vr0dc8meaabaqaciGacaGaaeqabaqabeGadaaakeaafaqabeGadaaabaGaemiuaaLaei4waSLaemiwaG1aaSbaaSqaaiabdMgaPjabcYcaSiabdQgaQbqabaGccqGHKjYOcqWGTbqBcqGGDbqxaeaacqGHKjYOaeaacqWGLbqzdaahaaWcbeqaaiabgkHiTmaaleaameaacqGGOaakcqWGfbqrcqGGBbWwcqWGybawdaWgaaqaaiabdMgaPjabcYcaSiabdQgaQbqabaGaeiyxa0LaeyOeI0IaemyBa0MaeiykaKYaaWbaaeqabaGaeGOmaidaaaqaaiabikdaYiabdweafjabcUfaBjabdIfaynaaBaaabaGaemyAaKMaeiilaWIaemOAaOgabeaacqGGDbqxaaaaaaGcbaaabaGaeyizImkabaGaemyzau2aaWbaaSqabeaacqGHsisldaWcbaadbaGaeGymaedabaGaeGOmaiJaeiikaGIaemyBa0Maey4kaSIaeGymaeJaeiykaKcaaaaakiabc6caUaaacqGHKjYOcqWGLbqzdaahaaWcbeqaaiabgkHiTmaaleaameaacqGGOaakcqWGTbqBcqGHRaWkcqaIXaqmcqGHsislcqWGTbqBcqGGPaqkdaahaaqabeaacqaIYaGmaaaabaGaeGOmaiJaeiikaGIaemyBa0Maey4kaSIaeGymaeJaeiykaKcaaaaaaaa@723D@

**Theorem 4. ***The iterative LP-relaxation algorithm gives a solution of O*(*m *ln *K*) *approximation*.

*Proof. *Suppose this algorithm runs for *t *iterations. The probability that all K(K−1)2
 MathType@MTEF@5@5@+=feaafiart1ev1aaatCvAUfKttLearuWrP9MDH5MBPbIqV92AaeXatLxBI9gBaebbnrfifHhDYfgasaacH8akY=wiFfYdH8Gipec8Eeeu0xXdbba9frFj0=OqFfea0dXdd9vqai=hGuQ8kuc9pgc9s8qqaq=dirpe0xb9q8qiLsFr0=vr0=vr0dc8meaabaqaciGacaGaaeqabaqabeGadaaakeaadaWcaaqaaiabdUealjabcIcaOiabdUealjabgkHiTiabigdaXiabcMcaPaqaaiabikdaYaaaaaa@337D@ inequalities in (5) are satisfied after *t *iterations is

(1−(e−1/2(m+1))t)K(K−1)2=(1−e−t/2(m+1))K(K−1)2≈e−K(K−1)2e−t/2(m+1).
 MathType@MTEF@5@5@+=feaafiart1ev1aaatCvAUfKttLearuWrP9MDH5MBPbIqV92AaeXatLxBI9gBaebbnrfifHhDYfgasaacH8akY=wiFfYdH8Gipec8Eeeu0xXdbba9frFj0=OqFfea0dXdd9vqai=hGuQ8kuc9pgc9s8qqaq=dirpe0xb9q8qiLsFr0=vr0=vr0dc8meaabaqaciGacaGaaeqabaqabeGadaaakeaafaqabeGadaaabaGaeiikaGIaeGymaeJaeyOeI0IaeiikaGIaemyzau2aaWbaaSqabeaacqGHsislcqaIXaqmcqGGVaWlcqaIYaGmcqGGOaakcqWGTbqBcqGHRaWkcqaIXaqmcqGGPaqkaaGccqGGPaqkdaahaaWcbeqaaiabdsha0baakiabcMcaPmaaCaaaleqabaWaaSqaaWqaaiabdUealjabcIcaOiabdUealjabgkHiTiabigdaXiabcMcaPaqaaiabikdaYaaaaaaakeaacqGH9aqpaeaacqGGOaakcqaIXaqmcqGHsislcqWGLbqzdaahaaWcbeqaaiabgkHiTiabdsha0jabc+caViabikdaYiabcIcaOiabd2gaTjabgUcaRiabigdaXiabcMcaPaaakiabcMcaPmaaCaaaleqabaWaaSqaaWqaaiabdUealjabcIcaOiabdUealjabgkHiTiabigdaXiabcMcaPaqaaiabikdaYaaaaaaakeaaaeaacqGHijYUaeaacqWGLbqzdaahaaWcbeqaaiabgkHiTmaaleaameaacqWGlbWscqGGOaakcqWGlbWscqGHsislcqaIXaqmcqGGPaqkaeaacqaIYaGmaaWccqWGLbqzdaahaaadbeqaaiabgkHiTiabdsha0jabc+caViabikdaYiabcIcaOiabd2gaTjabgUcaRiabigdaXiabcMcaPaaaaaGccqGGUaGlaaaaaa@71C3@

When *t *= 2(*m *+ 1) lnK(K−1)2
 MathType@MTEF@5@5@+=feaafiart1ev1aaatCvAUfKttLearuWrP9MDH5MBPbIqV92AaeXatLxBI9gBaebbnrfifHhDYfgasaacH8akY=wiFfYdH8Gipec8Eeeu0xXdbba9frFj0=OqFfea0dXdd9vqai=hGuQ8kuc9pgc9s8qqaq=dirpe0xb9q8qiLsFr0=vr0=vr0dc8meaabaqaciGacaGaaeqabaqabeGadaaakeaacqqGSbaBcqqGUbGBdaWcaaqaaiabdUealjabcIcaOiabdUealjabgkHiTiabigdaXiabcMcaPaqaaiabikdaYaaaaaa@363F@, the algorithm stops and returns a solution with probability *e*^-1^. Define *OPT*(*IP*) and *OPT*(*LP*) as the optimal solutions of the IP problem and the LP problem, respectively. Since the solution space of LP includes that of IP,

*OPT*(*LP*) ≤ *OPT*(*IP*).

Let the set of solutions returned in *t *iterations be {*Z*_1_, *Z*_2_,...,*Z*_*t*_}.

E[Z1]=E[∑k=1Nxk]=∑k=1Nyk=OPT(LP).
 MathType@MTEF@5@5@+=feaafiart1ev1aaatCvAUfKttLearuWrP9MDH5MBPbIqV92AaeXatLxBI9gBaebbnrfifHhDYfgasaacH8akY=wiFfYdH8Gipec8Eeeu0xXdbba9frFj0=OqFfea0dXdd9vqai=hGuQ8kuc9pgc9s8qqaq=dirpe0xb9q8qiLsFr0=vr0=vr0dc8meaabaqaciGacaGaaeqabaqabeGadaaakeaacqWGfbqrcqGGBbWwcqWGAbGwdaWgaaWcbaGaeGymaedabeaakiabc2faDjabg2da9iabdweafjabcUfaBnaaqahabaGaemiEaG3aaSbaaSqaaiabdUgaRbqabaGccqGGDbqxcqGH9aqpdaaeWbqaaiabdMha5naaBaaaleaacqWGRbWAaeqaaOGaeyypa0Jaem4ta8KaemiuaaLaemivaqLaeiikaGIaemitaWKaemiuaaLaeiykaKIaeiOla4caleaacqWGRbWAcqGH9aqpcqaIXaqmaeaacqWGobGta0GaeyyeIuoaaSqaaiabdUgaRjabg2da9iabigdaXaqaaiabd6eaobqdcqGHris5aaaa@5540@

Note that we repeat this algorithm only for those unsatisfied inequalities. Thus, *E*[*Z*_1_] ≥ *E*[*Z*_2_] ≥ ... ≥ *E*[*Z*_*t*_]. Let *x*_*p *_denote the final solution obtained in Step 4. The expected final solution is

E[∑p=1Nxp]≤E[∑p=1tZp]≤t×E[Z1]≤t×OPT(LP)≤2(m+1) ln K(K−1)2×OPT(IP)=O(m ln K)×OPT(IP).
 MathType@MTEF@5@5@+=feaafiart1ev1aaatCvAUfKttLearuWrP9MDH5MBPbIqV92AaeXatLxBI9gBaebbnrfifHhDYfgasaacH8akY=wiFfYdH8Gipec8Eeeu0xXdbba9frFj0=OqFfea0dXdd9vqai=hGuQ8kuc9pgc9s8qqaq=dirpe0xb9q8qiLsFr0=vr0=vr0dc8meaabaqaciGacaGaaeqabaqabeGadaaakeaafaqaaeqbdaaaaeaacqWGfbqrcqGGBbWwdaaeWbqaaiabdIha4naaBaaaleaacqWGWbaCaeqaaOGaeiyxa0faleaacqWGWbaCcqGH9aqpcqaIXaqmaeaacqWGobGta0GaeyyeIuoaaOqaaiabgsMiJcqaaiabdweafjabcUfaBnaaqahabaGaemOwaO1aaSbaaSqaaiabdchaWbqabaGccqGGDbqxaSqaaiabdchaWjabg2da9iabigdaXaqaaiabdsha0bqdcqGHris5aaGcbaaabaGaeyizImkabaGaemiDaqNaey41aqRaemyrauKaei4waSLaemOwaO1aaSbaaSqaaiabigdaXaqabaGccqGGDbqxaeaaaeaacqGHKjYOaeaacqWG0baDcqGHxdaTcqWGpbWtcqWGqbaucqWGubavcqGGOaakcqWGmbatcqWGqbaucqGGPaqkaeaaaeaacqGHKjYOaeaacqaIYaGmcqGGOaakcqWGTbqBcqGHRaWkcqaIXaqmcqGGPaqkcqqGGaaicqqGSbaBcqqGUbGBcqqGGaaidaWcaaqaaiabdUealjabcIcaOiabdUealjabgkHiTiabigdaXiabcMcaPaqaaiabikdaYaaacqGHxdaTcqWGpbWtcqWGqbaucqWGubavcqGGOaakcqWGjbqscqWGqbaucqGGPaqkaeaaaeaacqGH9aqpaeaacqWGpbWtcqGGOaakcqWGTbqBcqqGGaaicqqGSbaBcqqGUbGBcqqGGaaicqWGlbWscqGGPaqkcqGHxdaTcqWGpbWtcqWGqbaucqWGubavcqGGOaakcqWGjbqscqWGqbaucqGGPaqkcqGGUaGlaaaaaa@92D2@

With a high probability, the iterative LP-relaxation algorithm stops after *O(m *ln *K) *iterations and finds a solution of *O*(*m *ln *K*) approximation.     □

### An algorithm for finding auxiliary tag SNPs

This section describes an algorithm for finding auxiliary tag SNPs assuming robust tag SNPs have been computed in advance. Given a haplotype block *M*_*h *_containing *N *SNPs and *K *haplotypes, we define *C*_*tag *_⊆ *C *as the set of tag SNPs genotyped from a haplotype sample with some missing data. This haplotype sample may fail to be distinguished because of the ambiguity caused by missing data. We wish to find the minimum number of auxiliary tag SNPs from the remaining SNPs in the block to resolve the ambiguity. A formal definition of this problem is given below.

### Problem: Minimum Auxiliary Tag SNPs (MATS)

**Input: **An *N *× *K *matrix *M*_*h*_, and a set of SNPs *C*_*tag *_genotyped from a sample with missing data.

**Output: **The minimum subset of SNPs *C*_*aux *_⊆ *C *- *C*_*tag *_such that each pair of ambiguous patterns can be distinguished by SNPs in *C*_*aux*_.

The following theorem shows the NP-hardness of the MATS problem.

**Theorem 5. ***The MATS problem is NP-hard*.

*Proof. *Consider that all SNPs in *C*_*tag *_are missing. This special case of the MATS problem becomes finding the minimum tag SNPs from *C *- *C*_*tag*_, which is already known to be NP-hard [17]. Therefore, MATS is also NP-hard.     □

**Figure 11:**
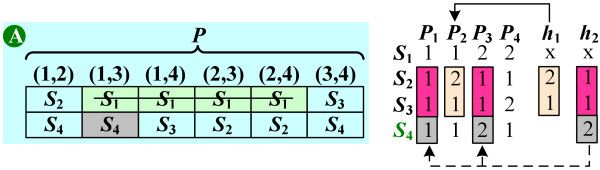
**An example of finding auxiliary tag SNPs**. The SNP *S*_1 _is missing and SNP *S*_4 _is the auxiliary tag SNP for *h*_2_. (A) The table that stores the set of robust tag SNPs.

Although the MATS problem is NP-hard, we show that auxiliary tag SNPs can be found efficiently when robust tag SNPs have been computed in advance. Without loss of generality, assume that these robust tag SNPs are stored in an (*m *+ 1) × |*P*| table *T*_*r *_(see Figure 11 (A)).

**Step 1. **The patterns that match the haplotype sample are stored into a set *A*. For example (see Figure 11), if we genotype SNPs *S*_1_, *S*_2_, and S_3 _for the sample *h*_2 _and the SNP *S*_1 _is missing, patterns *P*_1 _and *P*_3 _both match *h*_2_. Thus, *A *= {*P*_1_, *P*_3_}

**Step 2. **If |*A*| = 1, the sample is identified unambiguously and we are done (e.g., *h*_1 _in Figure 11). If |*A*| > 1 (e.g., *h*_2_), for each pair of ambiguous patterns in *A *(e.g., *P*_1 _and *P*_3_), traverse the corresponding column in *T*_*r*_, find the next unused SNP (e.g., *S*_4_), and add the SNP to *C*_*aux*_. As a result, the SNPs in *C*_*aux *_can distinguish each pair of ambiguous patterns, which are the auxiliary tag SNPs for the haplotype sample.

The worst case of this algorithm is that all SNPs in *C*_*tag *_are missing data, and we need to traverse each column in *T*_*r*_. Thus, the running time of this algorithm is *O*(|*T*_*r*_|) = *O*(*m*|*P*|).

## Authors' contributions

YTH and KMC design and implement the greedy algorithms. KZ and TC design and implement the iterative LP relaxation algorithm. All authors write and approve the manuscript.

## Supplementary Material

Additional File 1 **The program for the first greedy algorithm**. The Greedyl.zip file is compressed using WinZip and contains the JAVA source code for the first greedy algorithm. Click here for file

Additional File 2 **The program for the second greedy algorithm**. The Greedy2.zip file is compressed using WinZip and contains the JAVA source code for the second greedy algorithm. Click here for file

Additional File 3 **The program for the iterative LP-relaxation algorithm**. The ILP.zip file is compressed using WinZip and contains the Perl script for the iterative LP-relaxation algorithm. Click here for file
